# Legacy-making interventions in pediatric palliative care: A mixed methods systematic review

**DOI:** 10.1016/j.apjon.2025.100694

**Published:** 2025-03-28

**Authors:** Chanjuan Deng, Ruishuang Zheng, Jennifer Hong, Qiaohong Guo

**Affiliations:** a School of Nursing, Capital Medical University, Beijing, China; b Department of Hepatobiliary Cancer, Tianjin Medical University Cancer Institute and Hospital, Tianjin, China

**Keywords:** Children, Legacy-making intervention, Palliative care, Mixed methods, Systematic review

## Abstract

**Objective:**

To identify existing legacy-making interventions for children in the context of palliative care, to evaluate the reported outcomes of these interventions, and to explore the perceptions and experiences of children, family members, and healthcare professionals regarding these interventions.

**Methods:**

A mixed-methods systematic review was conducted. Six English databases, including PubMed, the Cochrane Library, Embase, CINAHL via EBSCO, ProQuest Nursing & Allied Health Database, and PsycINFO via EBSCO, and three Chinese databases, including CNKI, Wanfang, and Weipu, were systematically searched from inception to July 21, 2024. Articles reporting the results of qualitative, quantitative, or mixed-methods studies related to legacy-making interventions for children receiving palliative care were selected. A meta-synthesis and a quantitative narrative synthesis were conducted, and the findings were integrated using a convergent segregated approach.

**Results:**

Twenty-one articles were included. Existing legacy-making interventions were categorized into tangible legacy items and living legacy projects. These interventions were found to have numerous benefits, such as improving children's quality of life, preserving dignity, fostering adaptive coping mechanisms for illness-specific stressors for both children and their parents, enhancing parent-child communication, and promoting psychosocial well-being. They also contributed to reducing compassion fatigue and burnout among healthcare professionals. The majority of children's and parents' experiences with legacy-making interventions were positive, with high acceptability of these interventions. Suggestions from family members and healthcare professionals for enhancing these interventions were also reported.

**Conclusions:**

Legacy-making interventions could benefit children, their families, and healthcare professionals. More rigorous clinical trials should be conducted to confirm the effects of these interventions in the future.

**Systematic review registration:**

This systematic review has been registered on PROSPERO (Registration No. CRD42024490925).

## Introduction

Around 2.1 million children over the age of five die worldwide each year.^1^ The leading causes of death for these children are cancer and chronic diseases,^2^ resulting in physical, psychological, social, and spiritual suffering, especially in their end-of-life periods.^3^ Palliative care for children with life-threatening illnesses involves comprehensive care for the body, mind, and spirit, as well as support for their families.^4^ A significant aspect of palliative care for children is to address the psychosocial needs of children and their families,^5^ which could be identified or addressed by appropriate interventions, such as verbal expressions, symbolic play, gaming, art therapies, and legacy-making interventions.^5^ Among these, legacy-making interventions are crucial psychological interventions in pediatric palliative care, aiming at helping children and their families create lasting memories.^6^ Given the increasing importance of these interventions, there is a pressing need to systematically evaluate their scope, effectiveness, and impact, particularly within the realm of pediatric palliative care.

Legacy-making refers to actions, behaviors, or projects designed to create meaningful and lasting memories or products representative of dying children.^6^, ^7^, ^8^, ^9^ These interventions are usually facilitated by nurses, psychologists, social workers, or other interdisciplinary pediatric palliative care team members,^10^ who help children and families establish a legacy through abstract symbols or tangible objects,^11^ such as hand molds, handprints, memory books, photographs, videos, or other forms.^10^^,^^12^, ^13^, ^14^ In addition, specific legacy-making interventions have been developed or adapted specifically for dying children and their families, allowing them to create their own meaningful legacies. Examples of such interventions include dignity therapy and digital storytelling designed specifically for terminally ill children.^15^, ^16^, ^17^

The perceptions and experiences of terminally ill children and their families participating in legacy-making interventions have been studied. However, these findings can vary significantly among different children and families, influenced by the specific types of interventions and the cultural contexts in which they are situated.^18^^,^^19^ While most children and their parents considered legacy-making interventions enjoyable and meaningful,^19^ some experienced emotional suffering during the intervention due to feelings of regret in life, loss, and grief.^16^^,^^20^ In addition, the effects of legacy-making interventions have been evaluated using various methods and outcomes. Studies showed that these interventions had the potential to relieve the children's emotional distress, improve parent-child communication, and enhance the family's ability to cope with illness.^13^^,^^21^, ^22^, ^23^ Family members also reported that legacy-making interventions could help them cope with grief and maintain connections with their deceased children.^9^^,^^18^ However, some studies noted negative impacts associated with these interventions. For example, legacy objects created after a child's death brought negative experiences for parents.^20^

Several reviews have been conducted and provided valuable insights into legacy-making interventions across various contexts and populations, including the utility, feasibility, and benefits of legacy-making interventions as well as barriers to implementing such interventions. They also revealed significant research gaps, particularly the scarcity of studies focused on children and the absence of standardized practices in legacy-making interventions.^6^^,^^24^^,^^25^ To our knowledge, no systematic review has been done to synthesize evidence regarding legacy-making interventions targeted at children in pediatric palliative care.

Therefore, this systematic review aims to: (1) identify existing legacy-making interventions for children receiving palliative care, (2) evaluate the reported outcomes of these interventions, and (3) explore the perceptions and experiences of children, family members, and healthcare professionals regarding these interventions. We used a mixed methods systematic review approach, which allows for the integration of both quantitative and qualitative research findings. By combining these two types of data, the review can provide a more holistic understanding of the interventions' effectiveness, experiences, and contextual factors that influence their implementation and outcomes. This systematic review is expected to inform clinical practice of legacy-making interventions, and provide a direction for future research, ultimately contributing to improved psychosocial support for terminally ill children and their families.

## Methods

### Design

This systematic review was conducted following the Joanna Briggs Institute (JBI) methodology for mixed methods systematic reviews,^26^ and was reported according to the Preferred Reporting Items for Systematic Review and Meta-Analysis (PRISMA 2020) guidelines.^27^ The review protocol has been registered on PROSPERO (registration number: CRD42024490925).

### Search strategy

Six English databases, including PubMed, Cochrane Library, Embase, CINAHL via EBSCO, ProQuest Nursing & Allied Health Database, and PsycINFO via EBSCO, and three Chinese databases, including CNKI, Wanfang, and Weipu, were systematically searched from inception to July 21, 2024. An initial search of Google Scholar was undertaken to identify relevant studies, and keywords from the titles and abstracts of these studies, along with the index terms utilized to characterize these studies, informed the development of search strategies for PubMed. These strategies were then adapted for the other databases. Studies published in both English and Chinese were included, with no restrictions on publication dates. Detailed search strategies and results for each database can be found in Supplementary Material 1. Additionally, the reference lists of the included studies were manually screened for further relevant literature.

### Selection of studies

The inclusion and exclusion criteria for this mixed-methods systematic review were established based on the study objectives and through a systematic, structured process designed to ensure the relevance and quality of the included studies. The eligibility criteria were organized according to the PICOS framework, which includes population, intervention, comparison, outcome, and study type for quantitative studies, as well as population, phenomena of interest, and context for qualitative studies. The criteria are outlined as follows:

*Population:* Children under 18 years old receiving palliative care, excluding newborns and infants; family members of those children; and pediatric palliative care professionals involved in evaluating their perceptions of legacy-making interventions and the impacts of legacy-making interventions.

*Intervention:* Legacy-making interventions that were identified as actions, behaviors, or projects aimed at creating meaningful and lasting memories or products that can be beneficial for children and families both before and after children's death. These interventions were unrestricted, including memory books, photographs, videos, and similar others.

*Comparator*: No intervention, usual care, or waiting list control.

*Outcomes:* All reported outcomes of legacy-making interventions.

*Phenomena of interest:* Perceptions, experiences, or impacts of legacy-making intervention from children, family members or healthcare professionals.

*Context:* Hospice or palliative care for children, regardless of care settings, including inpatient, community-based or home care settings.

*Types of studies.* English- or Chinese-language peer-reviewed articles and dissertations of original qualitative, quantitative, and mixed methods empirical studies with full text available. Non-empirical studies, conference abstracts, and research protocols were excluded.

**Fig. 1:**
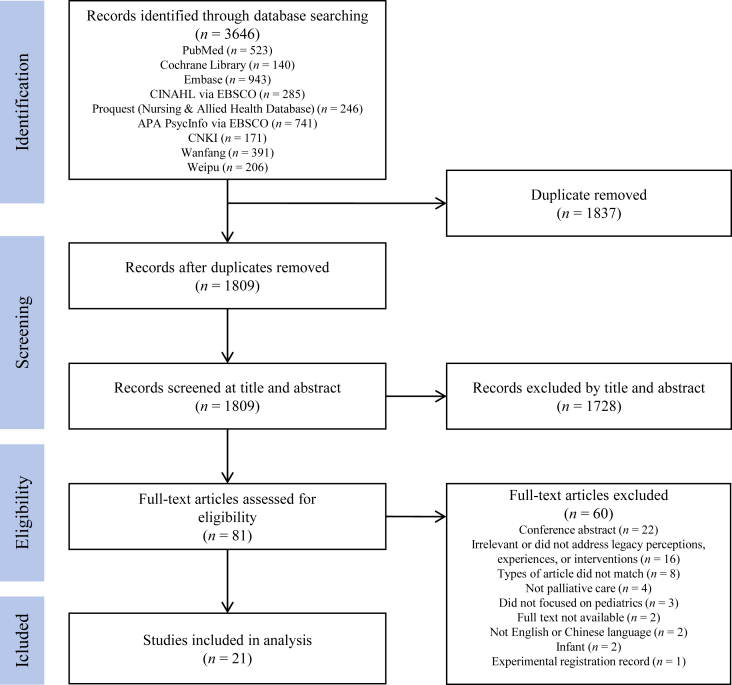
PRISMA flow diagram. PRISMA, Preferred Reporting Items for Systematic Reviews and Meta-Analyses.

All identified citations were collated and uploaded into Endnote X9 (Clarivate Analytics, PA, USA), where duplicates were removed. Two authors (CD and JH) independently screened the titles and abstracts to assess them against the inclusion and exclusion criteria, followed by a review of the full texts of the selected studies. Any disagreements between the two authors were discussed with the research team until a consensus was reached. The research team consisted of four members, including two nursing researchers with PhD degrees (RZ and QG) and two graduate nursing students (CD and JH). RZ and QG had extensive experience in palliative care research using different research methodology, and CD and JH received training in systematic reviews. The team held regular meetings to discuss progress and address challenges, ensuring rigor and consistency. The study selection process is presented in Fig. 1.

### Quality appraisal

The methodological quality of the selected studies was assessed using the JBI critical appraisal tools.^28^ The Mixed Methods Appraisal Tool (MMAT)^29^ was specifically employed to assess the quality of mixed methods studies. Details of the assessment results for each study are presented in Supplementary Material 2. Two authors (CD and QG) independently appraised the studies, and any disagreements were resolved through discussions within the research team until an agreement was reached. According to the JBI methodology, the decision to include a study can be based on meeting a pre-determined proportion of all criteria.^26^ To ensure the quality of the included studies while avoiding excessive exclusion, we selected studies using a 60% cutoff value, commonly used in previous studies, while also considering the relevance of the content.^30^^,^^31^

### Data extraction

Two authors (CD and JH) independently extracted quantitative and qualitative data using the JBI quantitative data extraction tool and the JBI qualitative data extraction tool.^28^ The authors, title, journal, year of publication, study methodology, method, phenomena of interest, setting, geographical location, participants, data analysis, and main results were extracted for qualitative studies and the qualitative component of mixed methods studies. The authors, title, journal, year of publication, study design, country, setting/context, year/time frame for data collection, the participants, the interventions (if applicable), the comparators (if applicable), the outcome measures, the condition and measurement method, the statistical analysis and main results were extracted for quantitative studies and the quantitative component of mixed methods studies. Qualitative data were extracted and organized into themes with illustrations, and the level of evidence for the findings was assigned as “Unequivocal,” “Credible,” and “Not Supported”.^32^ Any disagreements were discussed within the research team. The details of qualitative data were presented in Supplementary Material 3.

### Data synthesis and integration

This systematic review took a convergent segregated approach to synthesize and integrate the findings according to the JBI Manual for Evidence Synthesis.^26^ The convergent segregated approach involved a separate qualitative and quantitative synthesis, then the qualitative and quantitative syntheses were integrated. Quantitative data were pooled with a narrative summary instead of meta-analyses due to different outcome measures of included studies. Qualitative data were synthesized using the meta-aggregation approach,^32^ involving the synthesis of findings to generate a set of statements that represent the aggregation, through assembling the findings and categorizing these findings based on similarity in meaning. The process of meta-aggregation was presented in Supplementary Material 4. The findings of quantitative synthesis and qualitative synthesis were then configured according to the JBI methodology for mixed methods systematic reviews.^26^ In the integration process, three authors (CD, RZ and QG) independently conducted data analysis, compared the results, and discussed the differences and connections. Quantitative and qualitative evidence were juxtaposed and systematically organized to develop a coherent argument, resulting in a comprehensive configured analysis within the research team.^26^

## Results

### Study inclusion

This review encompassed 21 studies, including five qualitative studies,^9^^,^^18^^,^^20^^,^^33^^,^^34^ six quantitative studies,^13^^,^^21^, ^22^, ^23^^,^^35^^,^^36^ eight mixed-method studies,^10^^,^^12^^,^^14^, ^15^, ^16^, ^17^^,^^37^^,^^38^ and two case studies.^39^, ^40^^,^^40^

### Methodological quality

The quality scores were above 60% for all studies, and no study was excluded due to low quality. Among these studies, nine scored above 80%.^10^^,^^14^^,^^16^^,^^18^^,^^20^^,^^33^^,^^34^^,^^36^^,^^38^ The methodological assessment information is presented in Supplementary Material 2.

### Characteristics of included studies

Studies included in this review were published between 2009 and 2024. Among them, 18 were conducted in the USA,^10^^,^^12^, ^13^, ^14^^,^^17^^,^^18^^,^^20^, ^21^, ^22^, ^23^^,^^33^, ^34^, ^35^, ^36^, ^37^, ^38^, ^39^, ^40^ one was conducted in both the USA and Canada,^9^ one in China^16^ and one in Portugal.^15^ Of the five qualitative studies, four were descriptive studies,^9^^,^^18^^,^^20^^,^^34^ while one was a phenomenological study.^33^ Regarding the quantitative studies, four reported quantitative results from a randomized controlled trial (RCT) study, each focusing on different outcomes.^21^, ^22^, ^23^^,^^35^ Additionally, there was one cross-sectional study^13^ and one decedent cohort study.^36^ Of the quantitative component of the mixed methods studies, two were RCTs,^17^^,^^37^ one was a quasi-experimental study,^12^ and five were cross-sectional studies.^10^^,^^14^, ^15^, ^16^^,^^38^ The characteristics of all included studies are presented in Table 1.

**Table 1:** Characteristics of included studies.

Authors (year) and country	Aim/purpose	Design	Participants	Summary of key findings
Akard et al. (2015)^17^ USA	To examine the feasibility of a legacy-making intervention in children with cancer and the preliminary effects on outcomes related to quality of life	RCT using PedsQL v.4.0, Acute version, Follow-up child open-Ended questions and Parent survey	Children 7–17 years of age were diagnosed with any cancer and their primary parent caregiver (*n* ​= ​28). Intervention group (*n* ​= ​15) vs control group (*n* ​= ​13)	Feasibility was strong. The intervention group showed slightly better emotional and school functioning compared to controls. The children's digital story provided emotional comfort to them, facilitated communication between parents and children, and was a coping strategy for parents. The intervention helped children express their feelings, cope, and feel better emotionally.
Akard et al. (2020)^37^ USA	To describe the process of transforming a face-to-face format to a web-based legacy intervention for children with refractory or relapsed cancer and their parent caregivers	RCT using follow-up parent surveys	Children 7–17 years of age with relapsed or refractory cancer and their primary parent caregiver (*n* ​= ​98). Intervention group (*n* ​= ​61) vs control group (*n* ​= ​37)	Eighty-four child-parent dyads (85.7%) completed the intervention, and 81 (82.7%) completed survey questions. The web-based legacy intervention was feasible and acceptable, with parents perceived beneficial outcomes for the children, parents, and family.
Akard et al. (2021a)^35^ USA	To examine the impact of a web-based legacy intervention on dimensions of QOL among children with advanced cancer	RCT using PedsQL cancer module	Children 7–17 years of age with relapsed or refractory cancer and their primary parent caregiver (*n* ​= ​97). Intervention group (*n* ​= ​60) vs control group (*n* ​= ​37)	No statistically significant effect of the intervention for any of the primary QOL outcomes was found. However, legacy-making demonstrated small effects for improvements in child procedural anxiety and child perceived physical appearance compared to the wait-list control group.
Akard et al. (2021b)^23^ USA	To examine the impact of a web-based legacy intervention on parent-child communication	RCT using the Parent–Adolescent communication scale	Children 7–17 years of age with relapsed or refractory cancer and their primary parent caregiver (*n* ​= ​97). Intervention group (*n* ​= ​60) vs control group (*n* ​= ​37)	Tests of differences between the groups in the amount of change from pre-intervention to post-intervention revealed no statistically significant differences. The strongest effects were observed for improving open communication, decreasing child reports of problems with their mother, and overall communication.
Akard et al. (2021c)^22^ USA	To examine the effects of a legacy intervention for children with advanced cancer and their parents on parental coping strategies	RCT using responses to stress questionnaire	Children 7–17 years of age with relapsed or refractory cancer and their primary parent caregiver (*n* ​= ​97). Intervention group (*n* ​= ​60) vs control group (*n* ​= ​37)	None of the parent coping differences between the groups in change from pre-intervention to post-intervention was statistically significant. However, small to moderate effects in the direction of increased use of primary control and disengagement coping were noted.
Akard et al. (2021d)^38^ USA	To describe current legacy services offered by children's hospitals in the United States and compare with the previous results	Mixed-method cross-sectional design	Healthcare providers in teaching children's hospitals (hospitals, *n* ​= ​54)	All hospital offers legacy activities, with increased child participation compared to previous study. Patients receiving these activities include NICU patients, those with life-threatening traumatic injuries, prolonged life support, or hospice care. Slightly more hospitals now offer legacy-making to children with cancer, neurodegenerative diseases, or critical illnesses, though the increase is not statistically significant.
Andrews et al. (2020)^14^ USA	To better understand legacy making's effect on bereaved parents	Mix-method questionnaire survey	Parents of children who participated in the MTHS program (*n* ​= ​11 families, including 12 parents)	All respondents recommended the MTHS program to other families experiencing end-of-life decision-making. Parents heard about the program from pediatric palliative physicians and therapists such as music or child life. The respondents varied in how often they utilized their child's heartbeat recordings.
Cahalan et al. (2020)^39^ USA	To support legacy building as a medium for emotional healing prior to the end of life and to contextualize legacy-building projects using a case review	Case report design	A 17-year-old child named Thomas diagnosed with acute lymphoblastic leukemia	Thomas created a video journal, he had filmed over 30 video clips and edited them into a 36-min-long video montage. He and his mom tearfully watched the video together before going home to hospice. Additionally, thomas created a family plaster hand mold with his mother and brother when he was very ill during his most recent relapse hospitalization.
Cho et al. (2023)^21^ USA	To determine the effects of a digital storytelling-legacy intervention on the adaptive coping of children with recurrent or refractory cancer	RCT using response to stress questionnaire	Children 7–17 years of age with relapsed or refractory cancer and their primary parent caregiver (*n* ​= ​97). Intervention group (*n* ​= ​60) vs control group (*n* ​= ​37)	None of the differences in child coping between the groups in change from pre-intervention to post-intervention was statistically significant. The use of primary-control coping strategies increased and the use of disengagement coping strategies decreased in the intervention group relative to that of the control group although without statistically significant.
Daniels et al. (2024)^36^ USA	To explore differences in receipt of legacy-oriented interventions for children who died following treatment at a single, large, academic, pediatric cancer center, stratified by demographic factors and clinical factors	Retrospective decedent cohort study	Pediatric oncology patients of any age who were treated for a cancer diagnosis at a pediatric cancer center and died between 2015 and 2019 (*n* ​= ​678)	Fifty-two percent of patients received a legacy-oriented intervention. Older adolescents (≥ 13 years) were less likely to receive legacy-oriented interventions than younger ones. Patients with home/hospice deaths were also less likely to receive interventions compared to patients who passed away at research hospital locations. Hispanic patients and those in palliative care were more likely to receive interventions. No significant race association was noted.
Foster et al. (2009)^9^ USA and Canada	To explore bereaved parent and sibling perspectives of how children living with cancer created legacies	Descriptive qualitative design using semi-structured interviews	Bereaved parents or siblings (*n* ​= ​99)	Four major themes were identified: (1) many children living with cancer did and said things to be remembered, (2) some children living with cancer did not intentionally do or say anything to be remembered, (3) bereaved family members remembered deceased children, and (4) legacies provided inspiration for both bereaved family members and children living with advanced cancer.
Foster et al. (2012)^10^ USA	To examine health care provider perceptions of legacy-making activities offered by hospitals to pediatric patients and their families	Mixed-method cross-sectional design	Healthcare providers in teaching children's hospitals (hospitals, *n* ​= ​77)	Nearly all providers surveyed reported offering legacy-making activities to ill children and their families, with patients and families usually completing the activity together. Most activities were offered before a patient died and when cure was no longer being sought. Perceived outcomes included benefits to bereaved families and a tangible memento of their deceased child.
Hirsh et al. (2023)^12^ USA	To determine the feasibility and acceptability of using videography to alleviate the stress of anticipatory mourning in pediatric palliative care patients	Mix-method quasi-experimental study	Children with end-stage life-shortening conditions ages 8 years of age or older and their parent/guardian (*n* ​= ​8)	The study was judged to be technically feasible and to be acceptable to the participants. There was a non-significant trend suggesting that participants felt less certain of the video's helpfulness after making it than they were before. Patient participants felt less sad, less angry, and decreased anxiety after making the video compared with their feelings before the video. The final themes identified from the analysis of video recordings included gratitude, willing items to others, memories, death/dying, disease experience, and faith/spirituality.
Jones et al. (2023)^33^ USA	To explore the legacy perceptions and experiences of bereaved parents/caregivers, and to inform legacy-oriented interventions in pediatric palliative care	Moustakas' psychological phenomenology design using semi-structured phenomenological interviews	Bereaved parents/caregivers (*n* ​= ​17)	Participants described their legacy experiences and perceptions across three themes: (1) definitions of legacy: Traits and characteristics, impacts on others, enduring presence; (2) manifestations of legacy: Tangible items, experiences, traditions, rituals, altruism; and (3) factors perceived to affect legacy experiences: Healthcare experiences, characteristics of the child's death, individual grief processes.
Julião et al. (2020)^15^ Portugal	To adapt the adult Portuguese DT question framework for adolescents (DT-QF-Adol) (aged 10–18)	Mixed-method	Expert panel and adolescents aged 10–18 followed in the ambulatory psychology clinic (*n* ​= ​17)	There was 100% agreement on the final consensus version and defined age group (10–18 years old). Adolescents reported that the DT-QF-Adol was clear, unambiguous, and not difficult to answer. They assumed that this information could positively affect the way parents and friends saw and cared for them, permitting others to understand their concerns and preferences.
Leigh (2016)^20^ USA	To examine the impacts of legacy-building interventions on bereaved parents and siblings, specifically how legacy-building interventions facilitated by healthcare professionals impact bereavement for the immediate family	Descriptive qualitative design using semi-structured Interviews	Bereaved parents and siblings (*n* ​= ​16)	Four major parent themes emerged: (a) Introduction of legacy building, (b) experience of legacy building items: Positive experiences and negative experiences (negative association with the term legacy building and positive association with the term legacy building), (c) psycho-social care, and (d) maintaining connection. Four major sibling themes emerged: (a) Experiences with legacy-building items, (b) sibling grief, (c) psycho-social care, and (d) maintaining connection.
Lin et al. (2024)^16^ China	To develop a pediatric family-based dignity therapy (P-FBDT) protocol for terminally ill children and their families	Mixed-method expert survey	Pediatric oncology or pediatric palliative care experts (*n* ​= ​14)	The P-FBDT protocol was recognized as highly reasonable, and the P-FBDT interview guide was endorsed as important, acceptable, clear, comprehensive, and suitable to be used in pediatric palliative care practice in Chinese culture. Potential benefits, possible challenges, and practical considerations of the P-FBDT were also proposed.
Love et al. (2022)^34^ USA	To characterize bereaved parents' perspectives on the value of legacy activities; to describe parent recommendations for optimizing the provision of legacy activities by child life specialists and music therapists	Descriptive qualitative design using semi-structured Interviews	Bereaved parents (*n* ​= ​19)	Three core themes: (1) the value of legacy interventions: Meaning-making, continuing bonds, and affirmation of life; (2) the practical roles, uses, and functions of legacy items, and (3) best practices for offering legacy interventions: Communication, timing, and creativity.
Schaefer et al. (2019)^13^ USA	To examine the impact of legacy artwork on bereaved caregivers' psychological functioning and grief and to compare caregivers' perceptions of support provided by the hospital throughout their child's cancer journey between the intervention and control groups	Cross-sectional study using brief symptom Inventory-18, prolonged grief Disorder-13, and questions regarding supportive services	Caregivers of children ages 2 months–25 years old, who died from cancer between 2009 and 2014 at Children's hospital of Alabama (*n* ​= ​44) Legacy artwork group (*n* ​= ​25) vs control group (*n* ​= ​19)	There were no significant differences in psychological functioning among caregivers who participated in legacy artwork versus those who did not participate. However, caregivers who created legacy artwork with their child reported significantly fewer symptoms of prolonged grief and a greater perception of support from healthcare providers compared with caregivers who did not engage in this activity.
Schaefer et al. (2020)^18^ USA	To explore the legacy-making and grief experiences of bereaved parents who participated in legacy artwork with their child before his or her death from cancer	Descriptive qualitative design using semi-structured interviews	Bereaved parents (*n* ​= ​12) Healthcare providers (*n* ​= ​12)	Five key themes emerged: (1) legacy artwork fosters family bonding and facilitates open communication about the child's impending death; (2) it offers parents opportunities for life review and meaning-making; (3) after the child's passing, these artworks are often displayed in the parents' home, allowing them to find comfort in continuing their connection with their deceased child; (4) the artwork can help alleviate parents' grief following their child's death; (5) it may reduce compassion fatigue among healthcare providers by giving them a meaningful outlet to cope with the loss of their patients
Schuelke and Rubenstein. (2020)^40^ USA	To report a case series of dignity therapy modified for a pediatric palliative care population	Case series	Children and their families who were aware that death may occur soon and receiving care from the palliative care service (*n* ​= ​4)	Four children consented to the publication of their experience. All four participants reported that the intervention was acceptable and expressed gratitude for their final generativity document. No patient or family reported distress or negative effects from participation in dignity therapy.

RCT, randomized controlled trial; PedsQL, pediatric quality oflLife inventory; QOL, quality of life; NICU, neonatal intensive care unit; MTHS, Music Therapy Heart Sounds program; P-FBDT, pediatric family-based dignity therapy; DT, dignity therapy; DT-QF-Adol, dignity therapy question framework for adolescents

### Key findings

#### Types of legacy-making interventions

Two types of legacy-making interventions were identified: tangible legacy items^10^^,^^13^^,^^14^^,^^18^^,^^20^^,^^33^^,^^34^^,^^38^ and living legacy projects.^10^^,^^12^^,^^15^, ^16^, ^17^^,^^37^, ^38^, ^39^, ^40^ Tangible legacy items were created by hospital staff or the children and family, which can be seen or touched at any time, serving as tangible reminders of the deceased children.^39^ These included heartbeat recordings, initials written on personal belongings, fingerprint charms, paintings, and other legacy artworks or objects.^10^^,^^13^^,^^14^^,^^18^^,^^20^^,^^33^^,^^34^^,^^38^ Living legacy projects involve living children and/or families participating in an oral, written, or media-based legacy-making process, which allows for meaning-making in the context of terminal illness and provides autonomy and control for the children.^39^ This type mainly included the adapted versions of dignity therapy for children,^15^^,^^16^^,^^40^ and media-based legacy-making interventions such as creating the children's digital storytelling through video recordings or web-based legacy-making platform^12^^,^^17^^,^^37^^,^^39^ in this review. Details of these interventions are presented in Table 2.

**Table 2:** Details of the types of legacy-making interventions.

Type of legacy-making intervention	Article	Legacy-making intervention	Description
Tangible legacy items: Keepsake items created by hospital staff and/or families, giving tangible reminders of the deceased child.	Andrews et al. (2020)^14^	Music therapy Heart Sounds (MTHS) program	Music therapy recorded the child's heartbeat. A digital stethoscope (Eko CORE) that generates a phonocardiogram, a graphical representation of S1 and S2 heart sounds, was used to record children's heartbeats as they approached the end of life. The heartbeat was then overlaid to a song or voice recording or kept as a stand-alone file. Child life specialists and art therapists also helped the participating family create an artistic embellishment of the phonocardiogram. The family could use the phonocardiogram of their child's heartbeat in other artwork.
	Jones et al. (2023)^33^	Tangible items	The child's legacy was tangible items such as “tee ball trophies”, initials written on personal belongings, handprints, memorial tattoos, and even a Japanese maple tree.
	Love et al. (2022)^34^	Legacy items	Legacy items provided to participants included a fingerprint charm, handprint plate, tile, canvas, heartbeat song, hand or foot mold, painting or other art project, and a book.
	Schaefer et al. (2019)^13^	Legacy artwork	The child's handprints and footprints as well as their favorite things (e.g., colors and characters) were commonly incorporated into the artwork. Poems, quotes, and metaphors were also included as a way to promote meaning-making regarding the child's impending death.
	Schaefer et al. (2020)^18^	Legacy artwork	Bereaved parents' legacy artwork experience with their children included paintings, graffiti, collages, plaster sculptures, and wall murals.
	Leigh (2016)^20^	Legacy items	Parents' and siblings' experience of legacy items included hand/footprints, 3-D hand mold, memory boxes, photographs, keychains and other legacy objects.
	Foster et al. (2012), and Akard et al. (2021d)^10^^,^^38^	Legacy-making activities	Legacy-making activities that healthcare facilities offered included hand molds/handprints, a lock of hair, a memory book or journal, photography, art, and others.
Living legacy projects: Living children and/or families participated in a legacy-making progress, which allows for meaning-making and provides autonomy and control for the children.	Schuelke and Rubenstein. (2020)^40^	Dignity therapy (DT)	The process included a recorded interview, an editing session, and the creating of a generativity document that the dying children provide to their chosen loved one(s). Audio-recorded interviews were completed in the hospital setting such as at the patient's bedside or in a meeting room by DT therapist. Then the DT therapist transcribed the audio recording, edited the narrative draft, and created the generativity document. For younger preverbal or nonverbal children, a novel option of dignity therapy by proxy was used. Special artwork, pictures, or creative formatting that reflected the child's personality were included in the generativity document.
	Julião et al. (2020)^15^	Dignity therapy question framework for adolescents (DT-QF-Adol)	The DT-QF-Adol for Portuguese children aged 10–18 included nine questions. Children would make up his or her answers to these questions. This tool can potentially be considered a good addition to providing DT in pediatric palliative care.
	Lin et al. (2024)^16^	Pediatric family-based dignity therapy (P-FBDT)	A therapist-facilitated psychotherapeutic intervention centered on the child–family unit. Both children and their families are invited to engage in family interactions including conversations and creative activities in the therapeutic session which would be audio/video-recorded based on the participants' preferences. The final form of P-FBDT is a generativity entity, which includes a generativity document as the main body, supplemented with various memorable items such as crafts or photos if participants would provide any.
	Foster et al. (2012) and Akard et al. (2021)^10^^,^^38^	Legacy-making activities	Legacy-making activities that were offered by healthcare facilities, including writing (letters, poetry, etc.), songwriting/music, and video.
	Akard et al. (2015)^17^	A legacy-making intervention via digital storytelling	A digital storytelling intervention where scheduled child interviews were video-recorded, incorporating the child's favorite activities, locations, family members, or pets. Then the videographer used the video, the child's song selection, and approximately 12 photographs to create a digital story for each child aged 7–17. The final product could be viewed and distributed via computer.
	Akard et al. (2020)^37^	A web-based legacy-making intervention	The legacy-making intervention is based on a web program. Families created an online account on the web-based program, and then the child (aged 7–17) used the program to create his/her story. The web program included four major components that guided children to: (1) answer legacy questions about themselves and connectedness with others; (2) upload photographs; (3) upload video; and (4) upload music. Finally, the family would receive an electronic copy of the child's digital storyboard.
	Hirsh et al. (2023)^12^	End-of-life therapeutic videography	Children (aged 8 or older) were recorded at whichever location was most convenient for them by the researcher without other people presented for the videotaping. If children requested their parent/guardian's presence, that was allowed. Children began to make the video after being given instructions on how to make the video. The topics that children discussed in their video recordings included important people or things, feelings about dying, missed people or things, forgiveness, good and difficult times and memories, wishes, and saying good-bay. Children could say or do anything he/she wished on the video with no time limit.
	Cahalan et al. (2020)^39^	A collaborative living legacy project	A child (aged 17) made the video by himself. He created a video journal where he would capture a video clip of himself every day of his stem cell transplant admission. Content of the child's video includes saying the date, how many days until his stem cell transplant, how his pain and anxiety were that day, his feelings and fears surrounding his transplant process, expressing fears for his life and uncertainty about his resolve to “keep fighting”, all while choking back tears or actively crying during the filming sessions. His support team of child life specialist, music therapist, and visual arts specialist provided him with the tools needed to complete this project, including an iPad for daily video capturing and apps for video editing and music-making.

#### Quantitative evidence

##### Feasibility and acceptability of legacy-making interventions

High feasibility and acceptability of legacy-making interventions were reported. Akard et al.^17^ reported the feasibility of a digital storytelling legacy-making intervention, with a participation rate of 78%, a completion rate of 96%, and an endorsement rate of 100% by children. A study conducted by Hirsh et al.^12^ showed that videography was technically feasible in creating an end-of-life legacy, with 80% of the children completing the intervention and all outcome measures, and most patients felt “very glad” about the video recording. Julião et al.^15^ and Lin et al.^16^ examined the feasibility and acceptability of the adapted Dignity Therapy for terminally ill children in Portugal and China by surveying children and healthcare professionals. The study by Julião et al.^15^ showed that the Portuguese dignity therapy question framework (DT-QF) was easy for terminal children aged 10–18 to answer and could positively influence how their parents and friends perceived and cared for them.^15^ Lin et al.^16^ found that over 90% of healthcare professionals regarded the child and family questions in pediatric family-based dignity therapy (P-FBDT) as important, acceptable, clear, comprehensive, and culturally sensitive, and viewed the P-FBDT as a promising psychological intervention for terminally ill children and their families.^16^

##### Effects of legacy-making interventions

The effects of legacy-making interventions on children's quality of life, children's and parental adaptive coping with illness-specific stressors, parent-child communication, and psychosocial well-being were explored,^12^^,^^13^^,^^17^^,^^21^, ^22^, ^23^^,^^35^ but no significant differences were found between the intervention group and the control group on all measures.^17^^,^^21^, ^22^, ^23^^,^^35^ However, potential effects were noted in some dimensions of these measures. A web-based legacy-making intervention demonstrated small effects for improvements in child procedural anxiety and child perceived physical appearance (Cohen's *d* ​= ​0.28–0.35) in terms of children's quality of life;^35^ small to moderate effects were noted in some aspects of children's and parents' adaptive coping,^21^^,^^22^ including increased use of primary-control coping strategies (Cohen's *d* ​= ​0.21), decreased use of disengagement coping strategies by children (Cohen's *d* ​= ​−0.19),^21^ and increased use of primary control (Cohen's *d* ​= ​0.18) and disengagement coping strategies (Cohen's *d* ​= ​0.31) by parents.^22^ The strongest effects of legacy-making interventions on parent-child communication were observed in children reporting improved open communication (Cohen's *d* ​= ​0.33) and overall communication with fathers (Cohen's *d* ​= ​0.20), and decreased communication problems with mothers (Cohen's *d* ​= ​−0.20), as well as in parents reporting improved open communication (Cohen's *d* ​= ​0.21) and overall communication (Cohen's *d* ​= ​0.25).^23^ Legacy videography tended to help children feel less sad, less angry, and less anxious, although no statistical differences were found (*P*s ​> ​0.05).^12^ Families who participated in legacy artwork perceived greater support from the hospital and fewer symptoms of prolonged grief than those who did not participate (*P*s ​< ​0.05), despite no significant differences in psychological functioning being observed.^13^

##### Factors influencing children's participation in legacy-making interventions

Only one quantitative study^36^ has examined the factors that influence children's participation in legacy-making interventions. Daniels et al.^36^ conducted a death cohort review to explore differences in characteristics of children between those who received legacy-making interventions and those who did not. The results showed that boys were 1.62 times more likely to receive a legacy-making intervention compared to girls (*P* ​= ​0.003); younger children (≤ 5 years) had 1.73 times the odds of receiving interventions compared to older children (≥ 13 years) (*P* ​= ​0.07); children from international/US territories had 2.68 times the odds (*P* ​= ​0.001), and children living within 100 miles of the hospital had 4.50 times the odds (*P* ​< ​0.001) of receiving an intervention compared to children living more than 100 miles from the hospital within the US. Additionally, children enrolled in a palliative care program had 6.49 times the odds of receiving a legacy-making intervention than those not enrolled (*P* ​< ​0.001). Compared to children who died at home or in hospice, those who died at hospital had 27.36 times the odds of receiving a legacy-making intervention (*P* ​< ​0.001), while those who died at outside hospitals had 0.63 times the odds (*P* ​= ​0.072), and those who died at unknown locations had 0.26 times the odds (*P* ​< ​0.001).

#### Qualitative evidence

Four synthesized findings have emerged from meta-aggregation. The details of the findings and the meta-aggregation process are presented in Supplementary Materials 3 and 4.

##### Synthesized finding 1: Perceptions about legacy-making interventions

Both children and parents generally perceived legacy-making interventions positively. Children found these activities enjoyable; parents described participation in legacy-making interventions as a positive experience, and expressed positive comments and appreciation for these interventions, noting they were very helpful.^17^^,^^20^^,^^37^ Although healthcare professionals considered such interventions as a promising psychological intervention for terminally ill children,^15^^,^^16^ they identified several challenges in intervention implementation, such as handling emotional fluctuations experienced by children, families, and even therapists during the interventions, poor compliance of children due to immature cognitive development, and difficulties in engaging families who might struggle to accept their child's impending death.^16^

##### Synthesized finding 2: Legacy-making experiences of children and family members

In legacy-making interventions, children engaged in various activities such as making crafts, sharing personal belongings and writing letters,^9^ and they also recalled important memories and expressed gratitude of family members and friends.^12^ Additionally, children shared their thoughts about death and dying, life and illness experiences, and spiritual reflections.^12^ Parents displayed legacy items in their homes to maintain a bond with the deceased children,^18^^,^^20^^,^^34^ while also creating events or projects in memory of their children, and incorporating abstract symbols of their children's legacies, including the children's qualities, concerns for family, beliefs about an afterlife, and personal connections with hospital staff. These elements served as meaningful aspects in the legacy-making process, allowing parents to honor their deceased children's memories.^9^^,^^20^ However, a few families reported unpleasant experiences with legacy-making interventions. For instance, the legacies of the deceased children reminded siblings of their sorrowful pasts,^20^ and some parents experienced feelings of anger, regret, or grief during the legacy-making process, and some of them expressed disappointment with the quality of the legacy items.^20^

##### Synthesized finding 3: Impacts of legacy-making interventions

The benefits of the interventions for children included enhanced emotional expression, improved communication, strengthened parent–child relationships, and the maintenance of dignity. They also served as a means to prepare for their death while inspiring them to positively influence others' lives.^9^^,^^16^^,^^37^ Families stated that legacy-making interventions helped them make sense of their children's illness or death, allowing them to honor their children's legacy while preserving memories and maintaining connections with their deceased loved ones, ultimately supporting them in coping with grief.^9^^,^^14^^,^^16^^,^^18^^,^^20^^,^^34^^,^^37^ Additionally, healthcare professionals reported that providing legacy-making interventions helped reduce compassion fatigue and burnout.^18^

##### Synthesized finding 4: Suggestions for intervention improvement

Family members and healthcare professionals provided several suggestions for improving legacy-making interventions. First, they suggested integrating unique or special personal and family attributes into the legacy-making process to make these interventions more individualized and creative.^10^^,^^34^^,^^37^ For example, parents’ experiences with their children could guide the creation of their meaningful legacy items. Second, involving family members in the legacy-making process was suggested to create more opportunities for families to enjoy quality time together and engage in meaningful conversations.^20^^,^^37^ Third, it was advised to start the intervention earlier in the disease trajectory and offer it more frequently,^10^^,^^18^^,^^20^^,^^34^^,^^37^ although some parents preferred to receive the legacy-making intervention only when it was certain that the death of the child was approaching, fearing that early involvement in legacy-making might lead the child to lose hope for life.^34^ Fourth, improving the intervention protocol was recommended by clarifying intervention content, enhancing the user-friendliness of web-based legacy-making platforms, ensuring sufficient preparation, and allowing flexible execution.^16^^,^^37^ Fifth, establishing a well-trained multidisciplinary intervention team was considered essential.^10^^,^^18^^,^^20^^,^^38^ Finally, creating an online platform for families to share their experiences in legacy-making, and setting up evaluation criteria to assess the short- and long-term impacts of these interventions on children, families, and healthcare professionals, were also suggested.^37^^,^^38^

#### Integration of evidence

Both quantitative and qualitative evidence indicated that children and families generally had positive perceptions and experiences with legacy-making interventions. Quantitative findings highlighted a high level of acceptability of these interventions,^12^^,^^15^, ^16^, ^17^ which was further reinforced by qualitative evidence showing that children enjoyed the interventions and parents found them meaningful and beneficial.^17^^,^^20^^,^^37^ Although the quantitative data did not show significant differences in most measured variables,^12^^,^^13^^,^^17^^,^^21^, ^22^, ^23^^,^^35^ the qualitative insights revealed positive impacts of the interventions.^9^^,^^14^^,^^16^^,^^18^^,^^20^^,^^34^^,^^37^ Therefore, the qualitative findings supported and complemented the quantitative ones. The integration process is presented in Table 3.

**Table 3:** Integration process of quantitative and qualitative evidence.

Integrated results	Quantitative evidence	Qualitative evidence	Qualitative-quantitative evidence relationship
Children and families positively perceived and experienced legacy-making interventions	The completion rate and endorsement rate of digital storytelling were 96% and 100%,^17^ respectively; 80% of the participated children completed the videography intervention, and most of them reported feeling “very glad”;^12^ the Portuguese DT-QF was easy for terminal children aged 10–18 to answer;^15^ the questions in P-FBDT were perceived as important, acceptable, clear, comprehensive, and culturally sensitive.^16^	Children and parents were involved in various legacy-making activities, such as making crafts, expressing gratitude and missing loved ones, and displaying legacy items of deceased children in their homes.^9^^,^^12^^,^^18^^,^^20^^,^^34^ children found these legacy-making interventions enjoyable;^17^ parents expressed positive comments and appreciation for these interventions, finding they were very helpful and an amazing experience.^37^	Qualitative evidence supports quantitative evidence
Legacy-making interventions had positive impacts on children and families	Quality of life: No significant differences were found between the two groups, but web-based legacy-making intervention demonstrated small effects in procedural anxiety and perceived physical appearance (Cohen's *d* ​= ​0.35–0.28).^35^	Legacy-making interventions helped children enhance emotional expression, improve communication, strengthen parent–child relationships, maintain dignity, prepare for their death, and find inspiration to positively influence others' lives.^9^^,^^16^^,^^37^	Qualitative evidence complements quantitative evidence
	Coping: No significant differences were found between the groups, but web-based legacy-making intervention showed small effects on primary-control (Cohen's *d* ​= ​0.21) and disengagement coping strategies (Cohen's *d* ​= ​−0.19) among children.^21^	Legacy-making interventions helped families make sense of their children's illness or death, honor their children's legacy, preserve memories, maintain connections with their children, and cope with grief.^9^^,^^14^^,^^16^^,^^18^^,^^20^^,^^34^^,^^37^	
	Coping: No significant differences were found between the groups, but web-based legacy-making intervention showed trends toward increasing use of primary control (Cohen's *d* ​= ​0.18) and disengagement coping strategies (Cohen's *d* ​= ​0.31) in parents.^22^	Legacy-making interventions helped healthcare professionals reduce compassion fatigue and burnout.^18^	
	Communication: No significant differences were found between groups. The strongest effects of the web-based legacy-making intervention for children were observed in open communication (Cohen's *d* ​= ​0.33), overall communication with fathers (Cohen's *d* ​= ​0.20), and problems in communication with mother (Cohen's *d* ​= ​−0.20). For parents, the strongest effects were observed in open communication (Cohen's *d* ​= ​0.21) and overall communication (Cohen's *d* ​= ​0.25).^23^		
	Psychosocial well-being: Children trended feeling less sad, less angry, and less anxious after making the video, although no significant differences were found (all *Ps* ​> ​0.05);^12^ families perceived greater support from the hospital and fewer symptoms of prolonged grief (all *Ps* ​< ​0.05).^13^		

## Discussion

### Main findings

This mixed methods systematic review synthesized findings from 21 quantitative, qualitative, and mixed methods studies concerning legacy-making interventions for terminally ill children in pediatric palliative care to summarize the types of these interventions, their potential effects and impacts, and the perceptions and experiences of children, family members, and healthcare professionals.

A variety of legacy-making interventions have been developed to address the diverse needs of children receiving palliative care and their families. In this review, these interventions were classified into two types, tangible legacy items and living legacy projects, according to Cahalan's review.^39^ Tangible legacy items focus on "memory keeping",^41^ emphasizing their role in helping families maintain a connection with the deceased children and providing comfort after their loss. This type of intervention is particularly suitable for families who may find it challenging to express themselves deeply, such as young children or families with limited communication skills. Tangible legacy items are generally not constrained by language or culture, making them widely accepted and easily adaptable to diverse cultural contexts.^38^ Meanwhile, living legacy projects emphasize "meaning making", engaging children and families in active reflection and narrative processes that foster meaning-making, and enhance their sense of control and hope.^42^ Living legacy projects are better suited for children and families capable of reflection and self-expression. The two types of legacy-making interventions are not mutually exclusive and can be combined to address the multidimensional needs of children and families, including memory preservation, meaning-making, and emotional expression.^39^ This categorization provides a clearer understanding of the goals, characteristics, and potential mechanisms underlying different legacy-making interventions. Future research could further explore strategies for effectively integrating various types of legacy-making interventions to enhance their effectiveness.

This systematic review found that legacy-making interventions were both feasible and acceptable for terminally ill children and their families, aligning with the findings of a previous systematic review by Boles et al.^6^ For families, maintaining a connection with their deceased child is a fundamental spiritual need.^43^ Legacy-making interventions offer comprehensive, child- and family-centered support, enabling families to create lasting memories and sustain a bond with their deceased child.^16^ These characteristics contribute to the feasibility and high acceptability of these interventions among terminally ill children and their families. Notably, many legacy-making interventions have been culturally adapted, allowing families from diverse cultural backgrounds to participate more fully and derive meaningful benefits.^15^^,^^16^ Cultural diversity plays a crucial role in legacy-making interventions, as individuals from different cultural backgrounds may respond differently to the same approach. These cultural differences profoundly impact the feasibility, acceptability, and effectiveness of such interventions. When designing and implementing legacy-making interventions for children, it is important to consider their cultural perspectives on death, emotional expression, the social dynamics of parent–child relationships, and methods of remembering those who have passed away.^15^^,^^16^^,^^37^ For example, death and dying are often considered taboo in Chinese culture. Thus, when implementing legacy-making interventions for Chinese children and families, it is essential to adopt more subtle and indirect forms of expression, such as using symbolic items to convey emotions and memories instead of engaging in direct verbal discussions.^16^^,^^44^ Such adjustments ensure that these interventions align with the family's cultural values and practices, thereby enhancing their relevance and acceptance. Moreover, advancements in digital technology have significantly expanded the accessibility and implementation of legacy-making interventions, utilizing tools such as video recordings and web-based platforms.^12^^,^^37^ These digital approaches overcome barriers related to time and geography, offering greater convenience for children and families to participate in these interventions.

In this systematic review, the experiences of children and families participating in legacy-making interventions were generally positive, highlighting the beneficial impacts of these interventions. However, some families reported negative experiences that highlighted the potential limitations of legacy-making interventions. For instance, certain family members experienced anxiety and sorrow as these interventions evoked difficult emotions related to death and loss.^16^^,^^20^ Some families felt unprepared to create legacies for their children due to unpredictable prognoses, which led to emotional distress.^45^ Additionally, reminiscing about happy times could trigger emotional fluctuations such as intense grief and depression, particularly when contrasted with their current suffering and facing impending death and loss.^16^^,^^46^^,^^47^ These negative experiences underscore the complexity of implementing legacy-making interventions, which is significantly influenced by individual differences and family cultural backgrounds.^48^^,^^49^ On one hand, factors such as individual personality and coping strategies could result in vastly different responses to the same intervention.^48^ On the other hand, unresolved conflicts or underlying issues within the family may be triggered or even exacerbated during the intervention process, affecting the well-being of the child and the family.^49^ Nevertheless, these non-positive experiences are not always enduring. While initial exposure to legacy-making interventions may elicit negative emotions, families often come to find meaning in these interventions and recognize their value over time.^25^ Legacy-making interventions not only document and preserve memories but also create a safe space for family members to address their emotional needs. This process can reduce the risk of post-traumatic stress disorder and complicated grief among family members.^50^ Therefore, to minimize negative experiences and enhance the positive effects of legacy-making interventions in pediatric palliative care, more precise assessment strategies prior to intervention are recommended. For example, healthcare professionals could use observation, interviews, and assessment tools to identify families or individuals who may be more vulnerable,^51^^,^^52^ allowing for tailored support strategies that facilitate a smoother adaptation to the intervention.

The effectiveness of legacy-making interventions has been well-documented in adult populations.^53^ This systematic review suggests that legacy-making interventions may also provide effective psychosocial support for terminally ill children and their families, consistent with Boles et al's study.^6^ However, the quantitative studies included in this review did not find statistically significant effects on the outcomes measured. In both adult and pediatric populations, many studies evaluating legacy-making interventions reported non-significant results,^35^^,^^54^^,^^55^ which may be attributed to small sample sizes or less sensitive measures that fail to capture the nuanced and complex impacts of these interventions on participants' psychological and emotional well-being.^54^^,^^56^ Additionally, participants' negative experiences during these interventions may temporarily overshadow their benefits, which could also contribute to the lack of statistical significance in quantitative findings. Therefore, it is crucial to balance the limitation of legacy-making interventions with their potential therapeutic benefits. Providing additional psychological support during the intervention process may help alleviate negative experiences for children and family, enhancing the overall effectiveness of legacy-making interventions. Despite these challenges, the moderate effects observed in this review are noteworthy. Given the significant distress faced by terminally ill children and their families, even moderate effects could yield meaningful benefits for these participants, reflecting the potential of legacy-making interventions to address their complex psychosocial needs.^6^ Furthermore, unlike in adult populations, there is a notable lack of RCTs evaluating the effects of legacy-making interventions in children. This systematic review identified only two RCT studies conducted by the same research team,^17^^,^^35^ making a meta-analysis unfeasible and limiting the assessment of the overall effects of these interventions. Future studies should prioritize the design of high-quality, clinical trials to provide robust evidence for the effectiveness of legacy-making interventions for children in palliative care.

Additionally, the qualitative studies included in this review have consistently highlighted the multifaceted positive impacts of these interventions as reported by children, families, and healthcare professionals, offering compelling evidence of their multidimensional benefits. The quantitative evidence provided a broad perspective on the feasibility and potential benefits of legacy-making interventions, which were complemented by qualitative data that explored the emotional and social implications in terms of helping families cope with grief and enhancing parent-child communication. The qualitative evidence could further demonstrate the value of psychosocial interventions and enhance the interpretation of the results.^57^ The integrated data illuminated the impact of legacy-making interventions and revealed the underlying mechanisms by examining how these interventions influence individual emotional expression and family interactions. Future research should emphasize capturing participants' subjective feedback and refining qualitative data collection methods to improve intervention evaluation strategies.

### Implications for nursing practice and research

This review could provide valuable insights into legacy-making interventions. Legacy-making interventions should be tailored to the cultural contexts of children and families to enhance their feasibility and effectiveness, addressing the unique psychological and emotional needs of each child and family. Successful implementation of these interventions requires providers to be well-trained in intervention techniques and be capable of offering psychological support to both children and their families.

This review offers direction for future research. Given the lack of statistical significance in many quantitative variables and the substantial heterogeneity among them, future studies should focus on evaluating relevant clinical variables, such as families’ quality of life, anxiety, depression, and grief. Reliable and valid assessment tools, such as the Pediatric Quality of Life Inventory (PedsQL) and the Hospital Anxiety and Depression Scale (HADS), should be utilized to measure these variables. Additionally, developing and validating specific scales for these variables within the context of legacy-making interventions will be crucial. Conducting more rigorous clinical trials will also be essential to explore the potential benefits and long-term impacts of these interventions.

### Strengthen and limitations

This is the first mixed methods systematic review that specifically addresses legacy-making interventions in pediatric palliative care. It provides valuable evidence regarding the potential effects and impacts of these interventions on terminally ill children and their families, while synthesizing the experiences and perceptions of children, family members, and healthcare professionals involved in these interventions. However, this review has several limitations. Firstly, like all systematic reviews, it aims for comprehensiveness, but may not include all relevant studies due to limitations in the search strategies or databases. Future research could refine the search strategy and expand the databases to develop more robust summaries of the evidence. Secondly, the restriction to studies published in English and Chinese may have limited the inclusion of relevant research, potentially overlooking legacy-making interventions in other cultural contexts. Future studies should consider exploring these interventions in more comprehensive cultural and linguistic settings. Thirdly, since this review focuses specifically on pediatric palliative care, studies conducted in non-palliative care contexts were excluded, which means the findings cannot be generalized to those settings. Finally, the precision of the conclusions might be limited by the absence of a meta-analysis. More high-quality clinical trials evaluating the effects of legacy-making interventions in pediatric populations are needed.

## Conclusions

This systematic review summarized the effects and impacts of legacy-making interventions for children receiving palliative care and their families and synthesized the perceptions and experiences of children, families, and healthcare providers regarding these interventions. The findings indicate that legacy-making interventions are both feasible and acceptable with children and family members generally viewing the experience positively. These interventions offer a range of benefits, such as improving family relationships and communication, and enhancing psychosocial well-being. Recommendations from family members and healthcare professionals highlight key areas for innovation and enhancement in these interventions. Further research is needed to explore both the immediate and long-term effects of these interventions in pediatric palliative care and to identify best practices for addressing the legacy-making needs of terminally ill children and their families.

## CRediT authorship contribution statement

**Chanjuan Deng**: Conceptualization, Methodology, Data curation, Formal analysis, Writing original draft. **Ruishuang Zheng**: Conceptualization, Methodology, Data curation, Formal analysis, and Writing review & editing. **Jennifer Hong**: Data curation, Writing review & editing. **Qiaohong Guo**: Conceptualization, Methodology, Data curation, Formal analysis, Funding acquisition, and Writing review & editing. All authors have read and approved the final manuscript.

## Ethics statement

Not required.

## Data availability statement

Data used in this systematic review are from previously published studies, which have been cited. The data collected for this study is available in the manuscript as Table 1, Table 2, Table 3 and Supplementary Materials 1–4.

## Declaration of generative AI and AI-assisted technologies in the writing process

No AI tools/services were used during the preparation of this work.

## Funding

This research was supported by the Beijing Natural Science Foundation (Grant No. 7232001). The funder had no role in considering the study design or in the collection, analysis, interpretation of data, writing of the report, or decision to submit the article for publication.

## Declaration of competing interest

The authors declare no conflict of interest. The second author, Rushuang Zheng, and the corresponding author,Prof. Qiaohong Guo, are editorial board members of Asia–Pacific Journalof Oncology Nursing. The article was subject to the journal's standardprocedures, with peer review handled independently of Dr. Zheng, Prof. Guo and their research groups.
