# Supplementary material for: Legacy-making interventions in pediatric palliative care: A mixed methods systematic review
Source: Asia Pac J Oncol Nurs. 2025 Mar 28;12:100694. doi: 10.1016/j.apjon.2025.100694 (PMC12104642; doi:10.1016/j.apjon.2025.100694)
Supplement: Multimedia component 1 [file mmc1.docx]

Supplementary Material 1 – Search Strategy

Supplementary Material 2 – Methodological Quality of Included Studies

Supplementary Material 3 – List of Qualitative Findings with Illustrations

Supplementary Material 4 – Results of Meta-Aggregation of Qualitative Findings

**Supplementary Material 1 – Search Strategy**

**PubMed**

| **#** | **Query** | **Results** |
| --- | --- | --- |
| #1 | (((((((((((((((((((((((legacy[Title/Abstract]) OR ("legacy making"[Title/Abstract])) OR ("legacy intervention"[Title/Abstract])) OR ("legacy building"[Title/Abstract])) OR ("legacy activit*"[Title/Abstract])) OR ("legacy artwork"[Title/Abstract])) OR ("legacy action"[Title/Abstract])) OR ("legacy behavio?r"[Title/Abstract])) OR ("legacy service?"[Title/Abstract])) OR ("digital legacy"[Title/Abstract])) OR ("dignity therapy"[Title/Abstract])) OR ("dignity intervention"[Title/Abstract])) OR ("dignity program"[Title/Abstract])) OR ("hear my voice"[Title/Abstract])) OR ("life review"[Title/Abstract])) OR ("life stor*"[Title/Abstract])) OR ("life plan intervention"[Title/Abstract])) OR ("life completion intervention"[Title/Abstract])) OR (storytelling[Title/Abstract])) OR ("hand mold*"[Title/Abstract])) OR ("video recording"[Title/Abstract])) OR ("memory book"[Title/Abstract])) OR (biograph*[Title/Abstract])) OR ("generativity document"[Title/Abstract]) | 43576 |
| #2 | (((((((((child*[Title/Abstract]) OR (p?ediatric[Title/Abstract])) OR (adolescen*[Title/Abstract])) OR (teenage*[Title/Abstract])) OR (juvenil*[Title/Abstract])) OR (minors[Title/Abstract])) OR (youth?[Title/Abstract])) OR ("young children"[Title/Abstract])) OR ("young people"[Title/Abstract])) OR ("school-age*"[Title/Abstract]) | 2119880 |
| #3 | (((((((((((((((((Neoplasms[MeSH Terms]) OR (cancer[Title/Abstract])) OR (oncology[Title/Abstract])) OR ("end of life"[Title/Abstract])) OR (end-of-life[Title/Abstract])) OR (life-limit*[Title/Abstract])) OR ("palliative care"[Title/Abstract])) OR ("hospice care"[Title/Abstract])) OR ("advanced illness*"[Title/Abstract])) OR ("serious illness*"[Title/Abstract])) OR (bereave?[Title/Abstract])) OR (hospitalize?[Title/Abstract])) OR ("chronic NEAR/5 disease"[Title/Abstract])) OR (die[Title/Abstract])) OR (death[Title/Abstract])) OR (dead[Title/Abstract])) OR (dying[Title/Abstract])) OR (loss[Title/Abstract]) | 6488405 |
| #4 | #1 AND #2 AND #3 | 523 |

**Cochrane Library**

| **#** | **Query** | **Results** |
| --- | --- | --- |
| #1 | (legacy OR "legacy making" OR "legacy intervention" OR "legacy building" OR "legacy activit*" OR "legacy artwork" OR "legacy action" OR "legacy behavio?r" OR "legacy service?" OR "digital legacy" OR "dignity therapy" OR "dignity intervention" OR "dignity program" OR "hear my voice" OR "life review" OR "life stor*" OR "life plan intervention" OR "life completion intervention" OR storytelling OR "hand mold*" OR "video recording" OR "memory book" OR biograph* OR "generativity document"):ti,ab,kw | 3782 |
| #2 | (child* OR p?ediatric OR adolescen* OR teenage* OR juvenil* OR minors OR youth? OR "young children" OR "young people" OR "school-age*"): ti, ab, kw | 331649 |
| #3 | MeSH descriptor: [Neoplasms] explode all trees | 125512 |
| #4 | (cancer OR oncology OR "end of life" OR end-of-life OR life-limit* OR "palliative care" OR "hospice care" OR "advanced illness*" OR "serious illness*" OR bereave? OR hospitalize? OR "chronic NEAR/5 disease" OR die OR death OR dead OR dying OR loss):ti,ab,kw | 393675 |
| #5 | #3 OR #4 | 427434 |
| #6 | #1 AND #2 AND #5 | 140 |

**Embase**

| **#** | **Searched** | **Results** |
| --- | --- | --- |
| #1 | legacy:ti,ab,kw OR 'legacy making':ti,ab,kw OR 'legacy intervention':ti,ab,kw OR 'legacy building':ti,ab,kw OR 'legacy activit*':ti,ab,kw OR 'legacy artwork':ti,ab,kw OR 'legacy action':ti,ab,kw OR 'legacy behavio?r':ti,ab,kw OR 'legacy service?':ti,ab,kw OR 'digital legacy':ti,ab,kw OR 'dignity therapy':ti,ab,kw OR 'dignity intervention':ti,ab,kw OR 'dignity program':ti,ab,kw OR 'hear my voice':ti,ab,kw OR 'life review':ti,ab,kw OR 'life stor*':ti,ab,kw OR 'life plan intervention':ti,ab,kw OR 'life completion intervention':ti,ab,kw OR storytelling:ti,ab,kw OR 'hand mold*':ti,ab,kw OR 'video recording':ti,ab,kw OR 'memory book':ti,ab,kw OR biograph*:ti,ab,kw OR 'generativity document':ti,ab,kw | 39470 |
| #2 | child*:ti,ab,kw OR p?ediatric:ti,ab,kw OR adolescen*:ti,ab,kw OR teenage*:ti,ab,kw OR juvenil*:ti,ab,kw OR minors:ti,ab,kw OR youth?:ti,ab,kw OR 'young children':ti,ab,kw OR 'young people':ti,ab,kw OR 'school-age*':ti,ab,kw | 2767770 |
| #3 | 'neoplasms'/exp | 6365008 |
| #4 | cancer:ti,ab,kw OR oncology:ti,ab,kw OR 'end of life':ti,ab,kw OR 'life limit*':ti,ab,kw OR 'palliative care':ti,ab,kw OR 'hospice care':ti,ab,kw OR 'advanced illness*':ti,ab,kw OR 'serious illness*':ti,ab,kw OR bereave?:ti,ab,kw OR hospitalize?:ti,ab,kw OR ((chronic NEAR/5 disease):ti,ab,kw) OR die:ti,ab,kw OR death:ti,ab,kw OR dead:ti,ab,kw OR dying:ti,ab,kw OR loss:ti,ab,kw | 6668419 |
| #5 | #3 OR #4 | 9834206 |
| #6 | #1 AND #2 AND #5 | 943 |

**CINAHL via EBSCO**

| **Search ID#** | **Searched Terms** | **Results** |
| --- | --- | --- |
| S1 | AB legacy OR "legacy making" OR "legacy intervention" OR "legacy building" OR "legacy activit*" OR "legacy artwork" OR "legacy action" OR "legacy behavio?r" OR "legacy service?" OR "digital legacy" OR "dignity therapy" OR "dignity intervention" OR "dignity program" OR "hear my voice" OR "life review" OR "life stor*" OR "life plan intervention" OR "life completion intervention" OR storytelling OR "hand mold*" OR "video recording" OR "memory book" OR biograph* OR "generativity document" | 8843 |
| S2 | AB child* OR p?ediatric OR adolescen* OR teenage* OR juvenil* OR minors OR youth? OR "young children" OR "young people" OR "school-age*" | 583814 |
| S3 | AB neoplasms OR cancer OR oncology OR "end of life" OR end-of-life OR life-limit* OR "palliative care" OR "hospice care" OR "advanced illness*" OR "serious illness*" OR bereave? OR hospitalize? OR "chronic NEAR/5 disease" OR die OR death OR dead OR dying OR loss | 770526 |
| S4 | S1 AND S2 AND S3 | 285 |

**Proquest ([Nursing & Allied Health Database](https://www.proquest.com/nahs?accountid=38642))**

| **set#** | **Searched for** | **Results** |
| --- | --- | --- |
| S1 | [summary(legacy OR "legacy making" OR "legacy intervention" OR "legacy building" OR "legacy activit*" OR "legacy artwork" OR "legacy action" OR "legacy behavio?r" OR "legacy service?" OR "digital legacy" OR "dignity therapy" OR "dignity intervention" OR "dignity program" OR "hear my voice" OR "life review" OR "life stor*" OR "life plan intervention" OR "life completion intervention" OR storytelling OR "hand mold*" OR "video recording" OR "memory book" OR biograph* OR "generativity document")](https://www.proquest.com/recentsearches.recentsearchtabview.recentsearchesgridview.scrolledrecentsearchlist.checkdbssearchlink:rerunsearch/7C0B881451F44E09PQ/None?site=nahs&t:ac=RecentSearches) | [11,751](https://www.proquest.com/recentsearches.recentsearchtabview.recentsearchesgridview.scrolledrecentsearchlist.checkdbssearchlink_0:rerunsearch/7C0B881451F44E09PQ/None?site=nahs&t:ac=RecentSearches) |
| S2 | [summary(child* OR p?ediatric OR adolescen* OR teenage* OR juvenil* OR minors OR youth? OR "young children" OR "young people" OR "school-age*")](https://www.proquest.com/recentsearches.recentsearchtabview.recentsearchesgridview.scrolledrecentsearchlist.checkdbssearchlink:rerunsearch/48F7AE435F7C4555PQ/None?site=nahs&t:ac=RecentSearches) | [490,014](https://www.proquest.com/recentsearches.recentsearchtabview.recentsearchesgridview.scrolledrecentsearchlist.checkdbssearchlink_0:rerunsearch/48F7AE435F7C4555PQ/None?site=nahs&t:ac=RecentSearches) |
| S3 | [mainsubject.Exact("neoplasms")](https://www.proquest.com/recentsearches.recentsearchtabview.recentsearchesgridview.scrolledrecentsearchlist.checkdbssearchlink:rerunsearch/39A957A094674471PQ/None?site=nahs&t:ac=RecentSearches) | [18,766](https://www.proquest.com/recentsearches.recentsearchtabview.recentsearchesgridview.scrolledrecentsearchlist.checkdbssearchlink_0:rerunsearch/39A957A094674471PQ/None?site=nahs&t:ac=RecentSearches) |
| S4 | [summary(cancer OR oncology OR "end of life" OR end-of-life OR life-limit* OR "palliative care" OR "hospice care" OR "advanced illness*" OR "serious illness*" OR bereave? OR hospitalize? OR "chronic NEAR/5 disease" OR die OR death OR dead OR dying OR loss)](https://www.proquest.com/recentsearches.recentsearchtabview.recentsearchesgridview.scrolledrecentsearchlist.checkdbssearchlink:rerunsearch/AD260B236B1040CEPQ/None?site=nahs&t:ac=RecentSearches) | [639,984](https://www.proquest.com/recentsearches.recentsearchtabview.recentsearchesgridview.scrolledrecentsearchlist.checkdbssearchlink_0:rerunsearch/AD260B236B1040CEPQ/None?site=nahs&t:ac=RecentSearches) |
| S5 | S3 OR S4 | [646,235](https://www.proquest.com/recentsearches.recentsearchtabview.recentsearchesgridview.scrolledrecentsearchlist.checkdbssearchlink_0:rerunsearch/AB8C023674E346DEPQ/None?site=nahs&t:ac=RecentSearches) |
| S6 | S1 AND S2 AND S5 | [246](https://www.proquest.com/recentsearches.recentsearchtabview.recentsearchesgridview.scrolledrecentsearchlist.checkdbssearchlink_0:rerunsearch/C8FA84037E25446APQ/None?site=nahs&t:ac=RecentSearches) |

**[APA PsycInfo](https://bnu.yyttgd.top/,DanaInfo=www.proquest.com,SSL+psycinfo?accountid=8554) via EBSCO**

| **Search ID#** | **Searched Terms** | **Results** |
| --- | --- | --- |
| S1 | AB legacy OR "legacy making" OR "legacy intervention" OR "legacy building" OR "legacy activit*" OR "legacy artwork" OR "legacy action" OR "legacy behavio?r" OR "legacy service?" OR "digital legacy" OR "dignity therapy" OR "dignity intervention" OR "dignity program" OR "hear my voice" OR "life review" OR "life stor*" OR "life plan intervention" OR "life completion intervention" OR storytelling OR "hand mold*" OR "video recording" OR "memory book" OR biograph* OR "generativity document" | 33080 |
| S2 | AB child* OR p?ediatric OR adolescen* OR teenage* OR juvenil* OR minors OR youth? OR "young children" OR "young people" OR "school-age*" | 1005265 |
| S3 | AB neoplasms OR cancer OR oncology OR "end of life" OR end-of-life OR life-limit* OR "palliative care" OR "hospice care" OR "advanced illness*" OR "serious illness*" OR bereave? OR hospitalize? OR "chronic NEAR/5 disease" OR die OR death OR dead OR dying OR loss | 344957 |
| S4 | S1 AND S2 AND S3 | 741 |

**CNKI**

| **Search ID#** | **Searched Terms** | **Results** |
| --- | --- | --- |
| S1 | (SU%= "dignity" or SU%= "dignity therapy" or SU%= "dignity intervention" or SU%= "dignity care" or SU%= "dignity maintenance" or SU%= "life review" or SU%= "retrospective therapy" or SU%= "legacy" or SU%= "create legacy" or SU%= "make legacy" or SU%= "generativity document") AND (SU%= "children" or SU%= "juvenile" or SU%= "pediatric" or SU%= "adolescent" or SU%= "teenager" or SU%= "youth" or SU%= "young people") NOT (SU%= "cultural legacy" or SU%= "cultural heritage" or SU%=" intangible cultural heritage" or SU%= "world heritage" or SU%= "Olympic Heritage" or SU%= "heritage disputes" or SU%= "industrial heritage" or CLC="D9*" or CLC="DF*") | 171 |

**Wanfang**

| **Search ID#** | **Searched Terms** | **Results** |
| --- | --- | --- |
| S1 | (Subject:("dignity" or "dignity therapy" or "dignity intervention" or "dignity care" or "dignity maintenance" or "life review" or "retrospective therapy" or "legacy" or "create legacy" or "make legacy" or "generativity document")) AND (Title or abstract:("children" or "juvenile" or "pediatric" or "adolescent" or "teenager" or "youth" or "young people")) NOT (Subject:("cultural legacy" or "cultural heritage" or SU%=" intangible cultural heritage" or "world heritage" or "Olympic Heritage" or "heritage disputes" or "industrial heritage" or CLC="D9*" or CLC="DF*")) | 391 |

**Weipu**

| **Search ID#** | **Searched Terms** | **Results** |
| --- | --- | --- |
| S1 | (M=("dignity" or "dignity therapy" or "dignity intervention" or "dignity care" or "dignity maintenance" or "life review" or "retrospective therapy" or "legacy" or "create legacy" or "make legacy" or "generativity document") AND M=(Title or abstract:("children" or "juvenile" or "pediatric" or "adolescent" or "teenager" or "youth" or "young people")) NOT (M=("cultural legacy" or "cultural heritage" or SU%=" intangible cultural heritage" or "world heritage" or "Olympic Heritage" or "heritage disputes" or "industrial heritage" or CLC="D9*" or CLC="DF*")) | 206 |

**Supplementary Material 2 – Methodological Quality of Included Studies**

***Critical Appraisal using JBI Checklist for Qualitative Research***

| **Author (Year)** | **Q1** | **Q2** | **Q3** | **Q4** | **Q5** | **Q6** | **Q7** | **Q8** | **Q9** | **Q10** | **Total of ‘Yes’ in the article (%)** |
| --- | --- | --- | --- | --- | --- | --- | --- | --- | --- | --- | --- |
| Forter et al. (2009) | U | Y | Y | Y | Y | N | N | Y | Y | Y | 70.00 |
| Jones et al. (2023) | Y | Y | Y | Y | Y | N | N | Y | Y | Y | 80.00 |
| Leigh (2016) | Y | Y | Y | Y | Y | Y | N | Y | N | Y | 80.00 |
| Love et al. (2022) | U | Y | Y | Y | Y | Y | Y | Y | Y | Y | 90.00 |
| Schaefer et al. (2020) | U | Y | Y | Y | Y | Y | Y | Y | Y | Y | 90.00 |

Y: Yes; U: Unclear; N: No; N/A: Not applicable

Q1: Is there congruity between the stated philosophical perspective and the research methodology?

Q2: Is there congruity between the research methodology and the research question or objectives?

Q3: Is there congruity between the research methodology and the methods used to collect data?

Q4: Is there congruity between the research methodology and the representation and analysis of data?

Q5: Is there congruity between the research methodology and the interpretation of results?

Q6: Is there a statement locating the researcher culturally or theoretically?

Q7: Is the influence of the researcher on the research, and vice-versa, addressed?

Q8: Are participants, and their voices, adequately represented?

Q9: Is the research ethical according to current criteria or, for recent studies, is there evidence of ethical approval by an appropriate body?

Q10: Do the conclusions drawn in the research report flow from the analysis, or interpretation, of the data?

***Critical Appraisal using JBI Checklist for Randomised Controlled Trials***

| **Author (Year)** | **Q1** | **Q2** | **Q3** | **Q4** | **Q5** | **Q6** | **Q7** | **Q8** | **Q9** | **Q10** | **Q11** | **Q12** | **Q13** | **Total of ‘Yes’ in the article (%)** |
| --- | --- | --- | --- | --- | --- | --- | --- | --- | --- | --- | --- | --- | --- | --- |
| Akard et al. (2021a) * | Y | N | Y | N | N | U | Y | Y | N | Y | Y | Y | Y | 61.54 |
| Akard et al. (2021b) * | Y | N | Y | N | N | U | Y | Y | N | Y | Y | Y | Y | 61.54 |
| Akard et al. (2021c) * | Y | N | Y | N | N | U | Y | Y | N | Y | Y | Y | Y | 61.54 |
| Cho et al. (2023)* | Y | N | Y | N | N | U | Y | Y | N | Y | Y | Y | Y | 61.54 |

*******These articles are based on a same RCT study.

Y: Yes; U: Unclear; N: No; N/A: Not applicable

Q1: Was true randomization used for assignment of participants to treatment groups?

Q2: Was allocation to treatment groups concealed?

Q3: Were treatment groups similar at the baseline?

Q4: Were participants blind to treatment assignment?

Q5: Were those delivering treatment blind to treatment assignment?

Q6: Were outcomes assessors blind to treatment assignment?

Q7: Were treatment groups treated identically other than the intervention of interest?

Q8: Was follow up complete and if not, were differences between groups in terms of their follow up adequately described and analyzed?

Q9: Were participants analyzed in the groups to which they were randomized?

Q10: Were outcomes measured in the same way for treatment groups?

Q11: Were outcomes measured in a reliable way?

Q12: Was appropriate statistical analysis used?

Q13: Was the trial design appropriate, and any deviations from the standard RCT design (individual randomization, parallel groups) accounted for in the conduct and analysis of the trial?

***Critical Appraisal using JBI Checklist for Analytical Cross Sectional Studies***

| **Author (Year)** | **Q1** | **Q2** | **Q3** | **Q4** | **Q5** | **Q6** | **Q7** | **Q8** | **Total of ‘Yes’ in the article (%)** |
| --- | --- | --- | --- | --- | --- | --- | --- | --- | --- |
| Schaefer et al. (2019) | Y | Y | Y | Y | N | N | Y | Y | 75.00 |

Y: Yes; U: Unclear; N: No; N/A: Not applicable

Q1: Were the criteria for inclusion in the sample clearly defined?

Q2: Were the study subjects and the setting described in detail?

Q3: Was the exposure measured in a valid and reliable way?

Q4: Were objective, standard criteria used for measurement of the condition?

Q5: Were confounding factors identified?

Q6: Were strategies to deal with confounding factors stated?

Q7: Were the outcomes measured in a valid and reliable way?

Q8: Was appropriate statistical analysis used?

***Critical Appraisal using JBI Checklist for Cohort Studies***

| **Author (Year)** | **Q1** | **Q2** | **Q3** | **Q4** | **Q5** | **Q6** | **Q7** | **Q8** | **Q9** | **Q10** | **Q11** | **Total of ‘Yes’ in the article (%)** |
| --- | --- | --- | --- | --- | --- | --- | --- | --- | --- | --- | --- | --- |
| Daniels et al. (2024) | Y | Y | Y | Y | Y | Y | Y | Y | Y | Y | Y | 100.00 |

Y: Yes; U: Unclear; N: No; N/A: Not applicable

Q1: Were the two groups similar and recruited from the same population?

Q2: Were the exposures measured similarly to assign people to both exposed and unexposed groups?

Q3: Was the exposure measured in a valid and reliable way?

Q4: Were confounding factors identified?

Q5: Were strategies to deal with confounding factors stated?

Q6: Were the groups/participants free of the outcome at the start of the study (or at the moment of exposure)?

Q7: Were the outcomes measured in a valid and reliable way?

Q8: Was the follow up time reported and sufficient to be long enough for outcomes to occur?

Q9: Was follow up complete, and if not, were the reasons to loss to follow up described and explored?

Q10: Were strategies to address incomplete follow up utilized?

Q11: Was appropriate statistical analysis used?

***Critical Appraisal using JBI Checklist for Case Report***

| **Author (Year)** | **Q1** | **Q2** | **Q3** | **Q4** | **Q5** | **Q6** | **Q7** | **Q8** | **Total of ‘Yes’ in the article (%)** |
| --- | --- | --- | --- | --- | --- | --- | --- | --- | --- |
| Cahalan et al. (2020) | Y | Y | Y | N/A | Y | Y | N | Y | 75.00 |

Y: Yes; U: Unclear; N: No; N/A: Not applicable

Q1: Were patient’s demographic characteristics clearly described?

Q2: Was the patient’s history clearly described and presented as a timeline?

Q3: Was the current clinical condition of the patient on presentation clearly described?

Q4: Were diagnostic tests or assessment methods and the results clearly described?

Q5: Was the intervention(s) or treatment procedure(s) clearly described?

Q6: Was the post-intervention clinical condition clearly described?

Q7: Were adverse events (harms) or unanticipated events identified and described?

Q8: Does the case report provide takeaway lessons?

***Critical Appraisal using JBI Checklist for Case Series***

| **Author (Year)** | **Q1** | **Q2** | **Q3** | **Q4** | **Q5** | **Q6** | **Q7** | **Q8** | **Q9** | **Q10** | **Total of ‘Yes’ in the article (%)** |
| --- | --- | --- | --- | --- | --- | --- | --- | --- | --- | --- | --- |
| Schuelke and Rubenstein. (2020) | Y | N/A | N/A | Y | Y | Y | Y | Y | N | N/A | 60.00 |

Y: Yes; U: Unclear; N: No; N/A: Not applicable

Q1: Were there clear criteria for inclusion in the case series?

Q2: Was the condition measured in a standard, reliable way for all participants included in the case series?

Q3: Were valid methods used for identification of the condition for all participants included in the case series?

Q4: Did the case series have consecutive inclusion of participants?

Q5: Did the case series have complete inclusion of participants?

Q6: Was there clear reporting of the demographics of the participants in the study?

Q7: Was there clear reporting of clinical information of the participants?

Q8: Were the outcomes or follow up results of cases clearly reported?

Q9: Was there clear reporting of the presenting site(s)/clinic(s) demographic information?

Q10: Was statistical analysis appropriate?

***Critical Appraisal using Mixed Methods Appraisal Tool***

| **Author (Year)** | **Screening*** | | **Mixed method component** | | | | | **Qualitative component** | | | | | **Quantitative component** | | | | | **Total of ‘yes’ per article (%)** |
| --- | --- | --- | --- | --- | --- | --- | --- | --- | --- | --- | --- | --- | --- | --- | --- | --- | --- | --- |
|  | **S1** | **S2** | **Q**  **5.1** | **Q**  **5.2** | **Q**  **5.3** | **Q**  **5.4** | **Q**  **5.5** | **Q**  **1.1** | **Q**  **1.2** | **Q**  **1.3** | **Q**  **1.4** | **Q**  **1.5** | **Q**  **2.1** | **Q**  **2.2** | **Q**  **2.3** | **Q**  **2.4** | **Q**  **2.5** |  |
| Akard et al. (2015) | Y | Y | N | N | C | Y | Y | Y | Y | Y | Y | Y | Y | Y | Y | C | Y | 73.33 |
| Akard et al. (2020) | Y | Y | N | N | C | Y | Y | Y | Y | Y | Y | Y | Y | Y | Y | C | Y | 73.33 |
|  | **S1** | **S2** | **Q**  **5.1** | **Q**  **5.2** | **Q**  **5.3** | **Q**  **5.4** | **Q**  **5.5** | **Q**  **1.1** | **Q**  **1.2** | **Q**  **1.3** | **Q**  **1.4** | **Q**  **1.5** | **Q 3.1** | **Q 3.2** | **Q 3.3** | **Q 3.4** | **Q 3.5** |  |
| Hirsh et al. (2023) | Y | Y | C | N | C | Y | Y | Y | Y | Y | Y | Y | Y | Y | N | C | Y | 66.67 |
|  | **S1** | **S2** | **Q**  **5.1** | **Q**  **5.2** | **Q**  **5.3** | **Q**  **5.4** | **Q**  **5.5** | **Q**  **1.1** | **Q**  **1.2** | **Q**  **1.3** | **Q**  **1.4** | **Q**  **1.5** | **Q 4.1** | **Q 4.2** | **Q 4.3** | **Q 4.4** | **Q 4.5** |  |
| Akard et al. (2021d) | Y | Y | N | N | N | Y | Y | Y | Y | Y | Y | Y | Y | Y | Y | Y | Y | 80.00 |
| Andrews et al. (2020) | Y | Y | N | N | N | Y | Y | Y | Y | Y | Y | Y | Y | Y | Y | Y | Y | 80.00 |
| Foster et al. (2012) | Y | Y | N | N | N | Y | Y | Y | Y | Y | Y | Y | Y | Y | Y | Y | Y | 80.00 |
| Julião et al. (2020) | Y | Y | N | N | N | Y | Y | Y | Y | Y | N | C | Y | Y | Y | C | Y | 60.00 |
| Lin et al. (2024) | Y | Y | Y | N | N | Y | Y | Y | Y | Y | Y | Y | Y | Y | Y | Y | Y | 86.67 |

Y: Yes; N: No; C: Can’t tell

S1: Are there clear research questions?

S2: Do the collected data allow to address the research questions?

5.1: Is there an adequate rationale for using a mixed methods design to address the research question?

5.2: Are the different components of the study effectively integrated to answer the research question?

5.3: Are the outputs of the integration of qualitative and quantitative components adequately interpreted?

5.4: Are divergences and inconsistencies between quantitative and qualitative results adequately addressed?

5.5: Do the different components of the study adhere to the quality criteria of each tradition of the methods involved?

1.1: Is the qualitative approach appropriate to answer the research question?

1.2: Are the qualitative data collection methods adequate to address the research question?

1.3: Are the findings adequately derived from the data?

1.4: Is the interpretation of results sufficiently substantiated by data?

1.5: Is there coherence between qualitative data sources, collection, analysis and interpretation?

2.1: Is randomization appropriately performed?

2.2: Are the groups comparable at baseline?

2.3: Are there complete outcome data?

2.4: Are outcome assessors blinded to the intervention provided?

2.5: Did the participants adhere to the assigned intervention?

3.1: Are the participants representative of the target population?

3.2: Are measurements appropriate regarding both the outcome and intervention (or exposure)?

3.3: Are there complete outcome data?

3.4: Are the confounders accounted for in the design and analysis?

3.5: During the study period, is the intervention administered (or exposure occurred) as intended?

4.1: Is the sampling strategy relevant to address the research question?

4.2: Is the sample representative of the target population?

4.3: Are the measurements appropriate?

4.4: Is the risk of nonresponse bias low?

4.5: Is the statistical analysis appropriate to answer the research question?

* Screening questions are excluded from the computation of ‘yes’ per article.

**Supplementary Material 3 – List of Qualitative Findings with Illustrations**

Level of evidence: “U” for “Unequivocal” , “C” for “Credible” , “N” for “Not Supported”

| **Akard et al. (2015). Digital storytelling: an innovative legacy-making intervention for children with cancer. Pediatric Blood Cancer. 62(4):658-65.** | | | |
| --- | --- | --- | --- |
| Findings | | | Illustration |
| 1 | Children perceived the Digital Storytelling intervention as fun and enjoyable. (U) | | All children (n = 14, 100%) reported that they liked the digital storytelling activity, especially the music and photographs. One child said, *“They came to my house and let me make a video about me. I got to show them my pets, talk all about me and stuff I like. Now it’s on Facebook and I can watch it with my mom and friends whenever I want to. It was so much fun!”* |
| 2 | Children did not like some of things in the Digital Storytelling intervention. (N) | | Only a few children commented on things they did not like, such as one child who reported he/she did not like reading. Another child said he/she liked two of the photographs used but not the others, commenting that other people had selected the photographs. |
| **Akard et al. (2020). Transforming a face-to-face legacy intervention to a web-based legacy intervention for children with advanced cancer. Journal of Hospice & Palliative Nursing. 22(1):49-60.** | | | |
| Findings | | | Illustration |
| 3 | Parents expressed positive comments and appreciation for the legacy intervention. (U) | | One parent said, *“Awesome project. It’s bittersweet to document the child’s journey.”* |
| 4 | Parents perceived positive intervention benefits for both the children and their family who were involved in creating their child’s story, including emotional expression and communication benefits and parent-child relationship benefits. (U) | | Five parents praised the emotional expression and communication benefits for children and their family members. Examples included positive recognition of the sharing aspects of the program and of how the intervention motivated the children to share their experiences. One parent said, *“I believe it allows families to express their emotions, how treatment from this disease makes them feel. It allows the child to really evaluate how it makes them feel both emotionally and physically.”* Another parent shared, *“It’s helped this family realize some underlying issues.”*  Two parents reported liking how the intervention strengthened the parent-child relationship. Examples included bonding and quality time between parents and children in navigating the website, uploading pictures, and creating the storyboard. One parent reported, *“This project was really a neat way for [ill child] to express himself and do something he felt would help others. He loved picking out photos and thinking about his favorites and even though we were not entirely sure how to go about doing things, we worked together, and it really did create a bond.”* |
| 5 | Parents’ suggestions regarding the best timing for when to offer the intervention ranged from the beginning to end of the illness trajectory. (U) | | One parent suggested that the intervention *“be offered and available in the beginning of the journey for those parents that want to document as they go.”*  Another parent suggested the beginning because of too many side effects after treatment began: *“I believe that at the beginning is okay, but when it was offered to my son it was when he was fully into his treatment, and side effects didn’t let him function much to begin with ... now with him catching up on ‘life’ and school, he finds it hard to get back into it.”*  Some parents perceived later in the trajectory would be best. One parent said, *“After treatment ... gives time to reflect and open conversations.”*  Some parents felt timing should be determined based on each individual child: *“Hard to say, each situation is different. When the child is ready.”*  Parent most frequently suggested that the intervention be offered throughout the children’s cancer journey. *One parent said timing would be best “when they are going through the process of the illness.”*  One parent felt any point in the illness trajectory was a good time:*“I think given the opportunity, any time would benefit.”* |
| 6 | Parents liked the Web-Based intervention exactly as it was. (U) | | Comments included parent reports such as this: *“It was very helpful. I’m not sure what more they could add to make it better.” Another parent stated, “There is nothing I would change.”* |
| 7 | Parents suggested to involve family members more in future legacy intervention. (U) | | One parent said, *“Maybe also ask parent input on story.”*  Another parent suggested involving siblings: *“I think it would be nice to have somewhere where siblings can also participate. Our youngest son always feels left out of things that have to do with his brother’s illness.”* |
| 8 | Parents suggested that more should be taken into account to accommodate the individual needs of ill children in future legacy intervention. (U) | | One parent said, *“Younger children or some that are not feeling well when completing this may require some assistance to complete.”*  Another said, *“Some kids don’t understand what it means due to the chemotherapy and radiation damage.”* |
| 9 | Parents felt that the legacy intervention program should be lengthened to start sooner and last longer. (U) | | One parent said, *“I would have loved to have known about this in the beginning and keep being able to add to it as we go.”*  Another suggested they would have liked *“more time to cover more of their journey.”* |
| 10 | Parents desired a sharing or community component to allow the children to read others kids’ stories and increase their awareness of other patients. (U) | | Parents reported this might *“…help them communicate and understand what a cancer child goes through to bring awareness for other cancer kids and their families.”* |
| 11 | The most frequent suggestion from parents was to make the web program more user-friendly. (U) | | Parents had difficulty using the app on different devices and issues getting the program to work. One parent said, *“The app itself was not user friendly. I had difficulty myself with the app.”* Another parent shared, *“The ease of creating was a little difficult.”*  Technical difficulties included logging in to the website, uploading pictures, and editing posts. One parent said, *“It took me forever to be able to log in.”* Another said, *“We had technical problems. Also, it would be better to add pictures from device straight to [website] ... some kids don’t feel like taking selfies or pics at times ... but they may have pics on their devices already they can share.”* |
| 12 | Parents had suggestions for improved intervention content and design. (U) | | One parent suggested: “*I think some of the dropdown menu choices ... [are] too limited. Maybe choices to change backdrop colors, etc.”*  Another parent said, *“The questions were too vague. My daughter was confused about what to write. More detailed questions would help.”* |
| 13 | All parents were positive about the movie format of legacy intervention, specifically mentioning their appreciation for the coherent and automated flow of the entire story. (U) | | Parents reported that the cinematic feature made the digital story more meaningful to themselves and their ill child. Parents especially mentioned how they liked the music that played in the background. One parent said, *“The music made it more emotionally significant. I really liked it a lot.”*  Parents reported that their children preferred the movie format as well. One parent said, *“It made him feel special to be able to read his story and know that others will be able to see it also ...”* Other parents shared that the movie “gave her a starting point for talking” or their child “thought it was cool.”  Parents perceived that the movie feature was easy and simple to use, and 13 (81.3%) of parents said that the new format make it easier to share their child’s story with others. A few parents provided feedback for future improvements, including options to select or change the music, rotate photographs, select full screen, and alter the speed of the movie progression. |
| 14 | Parents perceived the new movie feature of legacy intervention facilitated family conversations. (U) | | Many parents reported how watching the movie caused them to remember and look back, such as one parent who said, *“We talked about how much has changed. It was really nice to look back.”*  Another parent said, *“I was able to have conversations with him that I otherwise wouldn’t have had.”*  Another participant shared, *“My husband and I laughed and shed a few tears watching this, just thinking about how much has changed, what we have gone through ... We talked about how we are so much stronger because of what we have endured together.”*  One parent shared how the movie triggered a conversation between her and her ill child as well as her spouse: *“[Ill child] and I talked about how the project made her feel about her cancer diagnosis and treatment ... My husband and I have had multiple conversations regarding the project and how [ill child]’s diagnosis has changed our lives and family goals.”*  A bereaved parent expressed, *“I told people who [deceased child] had mentioned in the story. Since he’s gone, it means so much more.”* |
| **Andrews et al. (2020). Legacy building in pediatric end-of-life care through innovative use of a digital stethoscope. Palliative Medicine Reports. 1(1):149-155.** | | | |
| Findings | | | Illustration |
| 15 | Families elaborated the heartbeat recordings were meaningful for preserving of memories with their child. (U) | | *[My daughter] was my center and we would lay and hold her close to me. The heartbeat recording allows me to live in that memory whenever I want to. Thank you for that endless gift.*  *I love having my son’s heartbeat in a song we always sang together.*  *Do anything we can to preserve the short time we had with him. Thank you all.*  *I will still be able to hear my son’s heartbeat even though he has passed.*  *It keeps her here with me.*  *I love having his heartbeat recording to remind me of the tiny heart that changed my life forever. Even though I don’t have my sweet [son], I will forever be able to listen to his heart beat.*  *Hearing my son’s heartbeat helps in my healing.* |
| **Foster et al. (2009). Bereaved parents' and siblings' reports of legacies created by children with cancer. Journal of Pediatric Oncology Nursing. 26(6):369-76.** | | | |
| Findings | | | Illustration |
| 16 | Many children living with advanced cancer did things to be remembered, including making crafts for others, willing away belongings, writing letters to loved ones, and giving special gifts. (U) | | *“She [20-year-old] gave me lots of stuff. She gave me like a bunch of old t-shirts because she loved t-shirts. Those are special.”*  *“One thing that me and her did before she passed... we talked about her belongings. And each one of her nurses was to have a specific toy that she had. And she told me, she says, ‘Mommy, you have to give it to them after I am gone. And they have to know that I wanted them to have this to remember me.’”*  *“Yeah, he [16-year-old] did [made] a cement stone, like a stepping stone. And he put sign language ‘I love you’ and he put like ‘sis’ on the bottom ... He did [one] for my mom, my dad, my grandma, and good friends of his.”*  *“We never had like the one moment to talk about that. But so, she [13-year-old] made these crafts ... flowers out of paper. We have that as a token of her. (Interviewer: So even though she didn’t do it purposely to be remembered by ...) No, no, no. But she just made it there [in the hospital] and she goes, “Okay, mommy, I thought you’d like one.” Or when her aunts would come or her cousins, she actually made some for them, too. (Interviewer: So she wasn’t aware that she was passing, but she made things for everybody?) Yeah. Yeah.”*  *“...All that I care for is that you remember me and don’t forget me.”-- an 11-year-old girl.* |
| 17 | Children did not intentionally do or say things to be remembered because of their illness, age, or unexpected death. Because some children with advanced cancer did not know they were dying, or family members think their child with cancer did not need to do or say anything to be remembered. (U) | | *“We didn’t know he [16-year-old] was going to pass away so quickly. When my mom was talking to him about making a will or whatever, he said he wasn’t ready for that. And she felt we could, that he could wait a little bit longer cause they thought he was gonna pass away at the end of the summer, but it ended up he sort of ended up passing away in the middle of the summer. And so because it’s so abrupt, he never actually told anybody, you know, about this and I want you to think of me when you look at this and that kind of stuff.”*  *“I don’t think he knew that he’d be going. I don’t think he realized. I don’t think he ever really realized he was really sick. I mean, it was just always a part of going to the hospital. He never really asked why his whole life. Yeah ... I don’t think he really realized that he was sick or anything.”*  *“You know we never discussed these things with (deceased child) ... We never talked about death and dying to him. And I didn’t want to. It was, I didn’t think it was appropriate ... I didn’t want him to have those thoughts. I didn’t want him to be scared. As it turned out, it happened suddenly, and maybe that was for a reason. I don’t think he ever knew that this was gonna take his life ... I don’t know that he ever thought about dying. I really can’t say with confidence that he did.”*  *“He [12-year-old] didn’t really want to talk about his death at all. He didn’t want to talk about it; he didn’t want to think about it. He just wanted to get on with doing whatever he was going to do that day ... I would ask him if he wanted to talk about anything or if he had any questions or anything, and he would always say “nope” and change the subject. So I mean certainly he is known for a lot of things, and I don’t think he’d done them with the intention of being remembered, but that’s how it evolved.”*  *“ I think she [14-year-old] was well aware of how deeply loved she was. So she didn’t need to leave anything behind.”*  *“I asked her [17-year-old] actually if there was anything that she wanted me to relay to anybody, and she said, ‘nope’ cause everybody knew it from her that she loved them ... She never wanted to be famous or anything, but she wanted to be remembered.”*  *“Before he died, he told me and his girlfriend and mom. He goes, ‘Before I die, I want to carry out a legacy or do something that nobody else has ever done.’ Then, 2 weeks later he goes, ‘You know, I have carried out a legacy. I’ve been like a dad to (sibling), and I’ve treated him like one more than the real dad did.’ And he goes, ‘I’ve already done what I needed to do.’”* |
| 18 | Bereaved family members remembered the deceased child. They remembered the qualities of the deceased child. (U) | | *“[He] touched a lot of lives in the hospital ... and has had a positive outreach to others ... reached people all around the world ... even with unspoken words.”*  Family members said their child was *“really a great kid”, or “such a funny kid”, or “had a ‘positive outlook’”, or “full of hope”.*  *“Unintentional was, to me, his [14-year-old] inner-strength. He faced it a lot better than I would have ... a load of courage.”*  *“There was a song ... It was the last thing she ever said and I remember ... ‘Let me see your grill’ ... and it says ‘smile for me daddy.’ Cause I got my gold [teeth] ... So every time I hear that song I always think about her ... music ... that was her passion ... The last thing she said to me ... she smiled and said, “Let me see your grill daddy.” That was the last thing she said to me before she stopped talking. She smiled to me and said that.”*  *“Yes, he had special requests ... he wanted to be buried underneath the tree in the backyard. We didn’t do that. He wanted to know if the hearse was gonna pick him up in a big car, and if it was, I suggested it was, he said, ‘Well, okay, well have them peel out for me cause I wanna peel out.’ So they peeled out of the drive. Yeah, crazy stuff.”* |
| 19 | Children’s concern for family was remembered by family members. (U) | | *“Momma it’s ok, it’s ok.”--an 8-year-old boy*  *“He [16-year-old] did ask me if I was going to be ok. So that was pretty helpful.”*  *“I love you.”-many children would say that.*  *“You know I love you. I really love you. Don’t you ever forget that.”--a 15-year-old boy*  *“The night before he [7-year-old] passed away ... he just told me he loved me and goodnight ...”*  *“She [4-year-old] said she loved me, and then I had to sing this crazy song for her.”*  *“He [15-year-old] was just concerned about us.”*  *“He [12-year-old] prayed a lot ... asking for blessing from God. For everybody. But one thing is so weird that he never included himself in that. He says that I ask God to have mercy on you, and Mom and Dad and everybody in the family, in hospital, all the people who worked and everything, but he never asked anything for himself.”* |
| 20 | Children’s beliefs about an afterlife was remembered by family members. (U) | | *“Nine days before she died, she told me that she was going to go be with Jesus soon … She said, ‘God’s put peace in my heart’.”*  *“She [3-year-old] told me not to worry about it, that she was going to make it all right with her friend. She went to Care-a-Lot Heaven. And knew she was going and told me that I would be there to meet her at the spot when it was my time to go. So she knew she was going. And I’ll never forget that. Being able to know that she would not forget me. Because she would be waiting for me at the spot. Knowing that, I knew she was going to be fine.”*  *“He [16-year-old] said, ‘I’m gonna go now, okay ... I’m gonna go to Heaven.’ ... he said he was gonna be okay.”*  *“I knew that she was really gone. She gave me a very big smile, so I know that wherever she is, she is okay and she was telling me that ‘Mom, its okay.’ That’s why I’m not worried. I know she’s okay and I know she wanted to be okay with whatever or however.”*  *“If I don’t come home, don’t feel sorry for me, be envious of me.”--a 17-year-old boy* |
| 21 | Children’s personal experience of living with cancer inspired the children to affect the lives of others. Cancer inspired children to prepare themselves for death or prepare others for their death. (U) | | *One mother recalled that her 16-year-old son “He really made a strong impact on a lot of people, because of his strong faith and trust in God to be in control and do what He knows is best for everybody.”*  *“I’ve never seen anyone that had an effect on so many people so young without any effort … I mean … she said in her letter ... she said, ‘... I’d like to be well but I am not. And if my sickness in any way causes someone to come to Lord, to get close to Lord, then it’s worth it all ...’”*  *“‘Mom, I want you to buy this for me,’ you know, her [deceased child], me, and (other friends) all their friends, ‘because they’re wings, it’s like angel wings for a Christmas present for them so we can all be angels.’”*  *“He said, ‘I won’t be back’ … And he’s like, ‘But I won’t have a chance to get macaroni and cheese again’ ... And he wanted me to sleep with him that night ...Maybe he knew.”*  *“She was only 2 [years old], but she and my wife had these necklaces ... Right before she went into surgery—(Deceased child)’s [necklace] said ‘Faith’ and (wife)’s [necklace] said ‘Strength.’ And (deceased child) made them change ... she [deceased child] wanted to trade.”* |
| 22 | While cancer inspired children with cancer to live their lives differently, their advice, in turn, inspired bereaved parents and siblings to live life differently in the moment. (U) | | *“He [17-year-old] was always telling me to work harder on things and do better.”*  *“Somebody [my niece] was complaining. He looked at her and goes, ‘(Name of niece), I’m dying. Do you hear me complaining?’ And, when I even think about complaining, I’ll stop myself.”*  *“References to God were evident in many messages about how to live life.”*  *“His statement was God ... He [ill child] said, ‘Jesus died on the cross for our sins, and I thank God for that.’ ... So that will always be a mark.”*  *“His legacy he left was, ‘Do you know God?’ He did this to everybody that he ever met ... He would get the conversation around to God.”* |
| **Hirsh et al. (2023). End of life therapeutic videography in pediatrics: feasibility and acceptability. Omega-journal of Death And Dying. 88(2):465-476.** | | | |
| Findings | | | Illustration |
| 23 | All children expressed gratitude to their families and/or friends in the legacy videography. (U) | | *“I want to say thank you to my brothers for helping take care of me over the years.”* |
| 24 | Some children gave instructions in the legacy videography about who should have some of their personal items after their death. (U) | | A 20-year old female said, *“something I would like to give to somebody would be these two prayer shawls I have and I say I’d like to give them to my mom because she’s always been there for me and um I just pray that she will take them and anytime she just needs a break and needs to think of me she can just crawl under them and say I love her and there I’ll be with her.”* |
| 25 | Children recalled positive memories in the legacy videography. (U) | | *“... we went to Disney World and it was fun. We stayed, we got free food, free ice cream, all the ice cream you can eat.”* |
| 26 | Children made comments related to death/dying, as well as to missing family, friends, or experiences in the legacy videography. (U) | | *“If I do die, I would say goodbye to all my family and friends and everybody.”* |
| 27 | Children described some of their disease experience as well as the stigma of disease in the legacy videography. (U) | | *“I’ve been in the hospital for one whole year and it’s so frustrating to just be in the hospital”*  *“…when I was in Walmart, there was a little kid in the cart and he standed up and said, ‘Hey Daddy, why does that kid have scars on his head?’ I didn’t want to say tumor, so I said, ‘What happened was, aliens abducted me’ and the kids were like, so freaked out.”* |
| 28 | Spiritual statements were made by some children reflecting spirituality as a source of comfort in the legacy videography. (U) | | *“... those people at home are watching me and praying for me and just hope for the best.”*  *“And I want ... to have the rest of my life better. I know you can do it, God, I know you can do it, I know you can fix me ... ”* |
| **Jones et al. (2023). "They were here, and they still matter": A qualitative study of bereaved parents legacy experiences and perceptions. Palliative Medicine. 37(8):1222-1231.** | | | |
| Findings | | | Illustration |
| 29 | Altruism helped bereaved parents /caregivers honor and carry on their child’s legacy. The experience of losing a child has helped them channel their grief into serving others. (U) | | Many participants shared how their child’s legacy served as inspiration *“to do good,” and a “calling to do more, to be more” (P9).*  *“Paying it forward,” or “doing anything you can to make a difference”. (P7)*  One mother whose son died by drowning expressed interest in creating a program to teach swim safety, to *“save somebody else’s life” (P12).*  Another mother created a foundation in her child’s honor to fund research on congenital heart disease and Down Syndrome, reflecting *“I feel like I get to continue on her legacy” (P9).*  (Channeling their grief into serving others) *“that’s part of that process, that’s part of his legacy, and it’s not something you can leave out” (P7).*  *“I understand how it feels to lose a child to various health problems. I don’t understand a lot of other things in this life, but I understand that. And I understand all of the different emotions. So that’s why I wanted to participate in this study thinking maybe down the road it will help somebody else.” (P14).*  *“I wish I could be more helpful, help people who have gone through something like this ... You live with a loss. There’s always a hole, but you find things to fill in the edges of the hole.” (P13)* |
| **Leigh. (2016). Handprints on the soul: the impact of legacy building interventions on bereaved families. Dissertation. California Institute of Integral Studies.** | | | |
| Findings | | | Illustration |
| 30 | Parents never being offered legacy building ,or not being explicitly told what legacy building was or the intended purpose for legacy building. They felt anger and regret for that. (U) | | *“Just to be clear, the whole concept of legacy planning was never ever introduced to us. So we had no idea about it, we only learned about it a few years after [his] death. So no one introduced it to us. But if we focused on the hand and foot prints, I think it was within the guise of, “here’s another art therapy kind of activity where let’s do hand prints, lets do foot prints, and growing kids change shapes and sizes.” So it makes kinda sense to do. But we never did that with the intention of saying “oh we need to plan [his] legacy or think it’s part of legacy planning so let’s capture his hand and foot prints.” “The only time it did occur was when he was basically dead, lying on the hospital bed.” (Timothy)*  *“If you had got me maybe four years ago, you probably would have seen a very angry upset person that no one really talked to me about this. And why didn’t they talk to me about these things ... I think I would have said I felt a lot of regret and no one talked to me about legacy building ... about the fact that here I would be burying my child ... and not knowing his wishes, desires, his outlook of things ya know or anything like that. And I held a lot of anger and guilt about that for a long time.” (Morgan)* |
| 31 | Parents wanted to have had the choice to participate in the creation of legacy building items if the opportunity would have been given to them. (U) | | *“Having options and choices and having this [legacy building] be a very organic process as opposed to “this is a legacy building thing or this is a handprint that we're doing because of that.” It becomes a very active and participatory experience for the whole family if we have options.” (Tonya)*  *“What would have helped me is if this was integrated into his care way before we knew he was dying. I would have preferred that much more, because then I think I would have had a much more different attitude about the hand and foot print.” (Morgan)* |
| 32 | It was important to offer legacy building sooner and more often throughout a child’s illness or hospitalization. (U) | | *“My advice would be to do things often and during. Not just at the end. Offer photos, offer pictures of them doing things and having fun and smiling” (Tonya)*  One father describes the importance of members of the psychosocial team bringing up the idea of legacy building at diagnosis: *“So when we turn to our psychosocial professionals who may be thinking about these things like legacy building and hand and foot prints, I think its sometimes important to remind us it’s an option that you may really like having, maybe not now, but down the road. And were not, but in either case you can toss them ... that would have helped me is if this was integrated into his care way before we knew he was dying.” (Morgan)* |
| 33 | The best person on the psychosocial team to initiate legacy conversations and also offer more opportunities for legacy building would be the child life specialist. (U) | | *“I wish they [child life] would have done more. I mean looking back we didn’t do tons, but I wish we would have done a lot more. If I would have known then what I know now, I would have really pushed crafts. Crafts and art and handprints and signatures and all that stuff. I think I would, people don’t think about it.” (Tonya)* |
| 34 | Legacy objects made after a child had died brought negative experience for parents. (U) | | *“The concrete ones were made when he was dead and they were holding up his foot and holding up his hands sticking it in concrete but he was dead and gone and really not participating ... And so while that was done its just ya know, too little too late. The concrete ones I haven’t dealt with [emotionally] ... in part because I think those remained at a time when he had just passed away and was lying on the bed dead. So I think of him not actually being involved technically, consciously that is, and him having passed away. Those things were made at that point in time. So, therefore it takes us back to that moment. which was perhaps one of the lowest points in my life.” (Timothy)*  *“The handprint that I was not involved in that [he] was not alive to do just does nothing for me ... it’s very impersonal. Its an object. If I had, had we made an afternoon of it per se, ‘ok we're gonna make a handprint lets goof around’ it would have been different. like arts and crafts, but it’s just a piece of plaster, there is no emotional attachment to it at all.” (Julia)* |
| 35 | Parents felt that the legacy building objects were not helpful, or harmful, for their grief, or not meaningful. The disappointment or negative experience was in the quality of the objects provided to them. (U) | | *“Those negative experiences just make me sad. I love that we have it, but to be honest, I haven't opened it a lot. Just right now its sort of painful.” (Devon)*  *“I don’t really look at them. Do I think it was helpful ... think I’m neutral about it to be honest.” (Morgan)*  *“I know where they are, they are in boxes still. But I never open them and I don’t look at them ... they are not particularly meaningful” (Morgan).*  *“If anything, the prints on the foam could have been done better. If I had known, I would say, ‘hey could you do them again?’ So that’s probably the only thing that was sad about the thing, because they kinda came out, they didn’t come out as well as I would have wished.” (Stewart)*  *“I absolutely treasure them but I wish they were made more, I wish they weren’t so fragile ... I’ve never touched them, I’m afraid to. I’m afraid to have anything happen to them. So they never leave that. I walk by and look at them but there is no touching them, there’s no putting your hands on them. Nothing like that. If they break they’re gone forever and I’m not sure I can take that kind of heartbreak. But I would love to have them in a situation where they are a little more durable.” (Tonya)* |
| 36 | Parents did not like the term “Legacy Building”. (U) | | *“Legacy building kinda sounds scary.” (Tonya)*  *“To me legacy is something that you pass on to your child and they pass it on to their children. But because he’s not here, I don’t really consider him part of a legacy” (Fiona)* |
| 37 | Parents found the legacy building items to be a positive experience, and reported finding great meaning in the objects. (U) | | *“ It’s mostly positive. I think people get confused and think you will automatically, you’ll go to a negative place with it. I think our brain is wired to want to go to the happy place first ya know. I think the pictures are very important and meaningful. It’s nice to have the ceramics and I don’t think that’s always an option but I mean I’m really glad I was able to figure that out ... I'm super glad I have that.” (Susan)*  *“The model magic ones were made when he was alive and vibrant, relatively speaking. He was still going thorough treatment and surgeries, but he was still very much a part of the process. And so those are those memories of that and when you look at those ... besides being bitter sweet, it’s not only remembrance of [him] and the fact that he's no longer with us. But in part, we remember making those with him, so therefore part of him is still there.” (Timothy)* |
| 38 | The objects provided and made by hospital staff were infused with significantly less meaning compared to objects made by family or friends, or bought or created by the parents themselves in remembrance of their child. (U) | | *“I think they are helpful, the photographs. The keepsakes from the hospital I just leave them in the box. For me its more helpful for the keepsakes that I have created, or bought to memorialize him.” (Ted)*  *“One of my best friends gave me this pendant with his name and birth and death date on one side. Its a heart. and on the other side are these little footprints ... I wear it a lot.” (Fiona).* |
| 39 | One meaningful aspect of legacy building was opportunities to continue their child’s legacy and memory after their death. (U) | | *“And another iteration of legacy building, is we do the kindness project that I do for him. Or the walk. Those are ways that I keep [him] with me that I have found helped me. The same way going back to [the house], and going to events, that’s my [son] time. That’s my way of keeping him in my life.” (Helena)*  *“We created a foundation two months after he passed away called the (child’s name) foundation ... And by having that foundation then trying to address the psychological, social, support needs for children and their families, we in part not only have built but maintain and enculturate and nurture a legacy that has albeit weak -- an albeit weak link to [our son].” (Timothy)* |
| 40 | For some families, the most meaningful aspect of legacy building is in the events or projects that they create in memory or honor of their child. (U) | | *“I created a memorial at his school...every year this is a large 5K. I also do St. Baldricks… So his legacy and there again I mentioned all of his friends who have gone on to college to do things in the medical field and that’s all because of him… And I get text messages on mother’s day, his birthday, his anniversary. His friends have really gone out of their way to stay in touch with me…I think perhaps whether you do it in the hospital or your bereavement groups after, I went ahead and put together a photo album of our time in the hospital. Which I think some parents may find helpful and useful.” (Julia)* |
| 41 | One further aspect of legacy building was the personal connections made with hospital staff while the child was alive. (U) | | *“The manger of child life said to me, talking about [my daughter] ‘that one I didn’t think she was going to make it’. The child life specialist said she earned ‘her wings’ with [my daughter]. She said she was there because of [my daughter] she was thinking of leaving her job but it was because of [my daughter] she stayed ... then 2–3 years after camp and after [my daughter] had died, the child life volunteer that worked with her pulls out her keychain and she's got that ring on her keychain that she carries with her every day. And she’s like ‘I look at this every day and think of her and it reminds me why I’m doing this’. And to hear that I’m like, how did this little girl inspire all these people?” (Kathy)*  *“So she learned a lot from him and one of the other moms in my group, her son was in the same hospital after [my son was] and the child life specialist had told me that she was thankful of the time she had with [my son]. She was able now to apply it to the other teenage boy and she wasn’t so afraid of dealing with a teenage boy.” (Julia)* |
| 42 | The term “Legacy Building” wad comforting and reflected parents’ positive experiences. (U) | | *“I think that's’ a good name because memory reminds you what was. My first thought was maybe something with memory because that’s what it’s for. But I think legacy handles it much better. Memory is kinda in the past if that makes sense. I think its aptly named. I wouldn't change it.” (Stewart)* |
| 43 | Parents remembered their child through touching objects, having legacy building items visible in the home, and through the public and private ways they have honored their child’s life. (U) | | *“I think it [staying connected] continues on two levels. There’s the private and the public. Privately ... its woven with grief and having certain prompts, props, and memories are what I have left now that [he] is gone and that’s sort of private. Publicly, we have the foundation ... I look for that kind of connection every morning coming down the staircase. There is a pic of him and I touch that in the morning and the evening when I come back upstairs ... And then, there are two places one in the morning as I’m heading to work and one in the evening as I’m heading home where I talk to him.” (Timothy)* |
| 44 | Tangible items created by the child, or created with the child, were important as representing a proof of life. Tangible items kept the relationship and connection in the present as opposed to the past. (U) | | *“Having these things here, it kinda solidifies that she was here. And she did matter”. (Kathy)* |
| 45 | Parents’ relationship to the legacy objects and items has shifted and changed over the course of their grief. (U) | | *“It’s just in our memory and the things that we did get to salvage ... I’m sure that box will be used for that at some point. So it was helpful at first but now it’s just there. So I don’t go back to it. At some point I will and so I’m glad I have it. But as far as the grief process, because I haven’t utilized it except at the very beginning. I don’t think it was, for me, that necessary. However, in 10 years maybe I’m still grieving and can really look at it and enjoy it then I would say yeah. So probably not helping with my grief but it’s something that I want and I can use down the road.” (Stewart)*  *“At that time maybe you're not ... some people might feel they don’t even want [legacy building items] or anything. But later on they might realize they might be happy they actually have it. I'm glad that they did too ... So I think that’s why right now its hard for me to look at them. Like, when we first got back from the hospital I would look at it but during that time too you're pretty numb. But now I can feel more, so I haven't looked at them ... for awhile I had it out on our dining table where we could see it every day. But now we have it in a box. ”(Fiona)* |
| 46 | Parents’ therapeutic and personal relationship with various members of the medical team influenced the memory that parents had to the legacy items. (U) | | *“I think it’s the only thing that would matter to me [who introduced legacy building] is how it was approached in terms of who is asking to. So if it’s a person you may have had more interactions with ... I think it really helps if whoever is approaching about the hand and foot print if someone has a relationship and care and the treatment process with the child and the family…When we turn to our psychosocial professionals who may be thinking about these things like legacy building and hand and foot prints, I think it’s sometimes important to remind us it’s an option that you may really like having, maybe not now, but down the road. And were not, but in either case you can toss them.” (Morgan)* |
| 47 | It was important for parents to have the opportunity to create legacy building items while their child is alive. (U) | | *“I think it’s absolutely essential that parents have an opportunity to do something like that. Because it’s a finite engagement you have with your child and nothing’s going to erase or hide nor lessen the memories you have of the pain and suffering and the subsequent loss and death of your child. But having something that’s there that is a reminder, that it’s a refection that was gone at a time and manner ... you know helps make it a little less worse. So I strongly recommend and support all parents having that opportunity to do that.” (Timothy)*  *“What would have helped me is if this was integrated into his care way before we knew he was dying. I would have preferred that much more because then I don’t think I would have had a much more different attitude about the hand and foot print.” (Morgan)* |
| 48 | Looking at the legacy building items brought back positive memories for siblings. (U) | | *“It makes me happy because it reminds me of all the things that he left for me. He left that bunny. He left my mom’s bunny Nibbles, and dad’s bunny Brian, and my bunny Nicky”. (Katie*  *“Yeah my memories are positive of making it.” (Derrick)* |
| 49 | Siblings thought it’s important to have the opportunity for the whole family to make legacy building items. (U) | | *“I think its good for people to make this because it kinda makes it kinda easier ... it can help people remember all the good memories”* |
| 50 | Siblings disliked looking at items such as photos or tangible reminders of their sibling. (U) | | *“I would have feelings of what I went through, if I really get into it and just start really thinking about him I cry ... I’ve gone through a lot of things and like it just reminds me of the past. “(Michael)* |
| 51 | Legacy building items helped siblings cope with and express their grief. (U) | | *“The handprints and pictures helped the grief. I feel like it did ... it kinda like calms me down”. (Katie)* |
| **Lin et al. (2024) Development of the pediatric family-based dignity therapy protocol for terminally ill children (ages 7-18) and their families: A mixed-methods study. Palliative & Supportive Care. 14:1-9.** | | | |
| 52 | P-FBDT could facilitate dignity conservation for terminal children. (U) | | *“It could potentially help us to know the real needs of children, especially the older ones who have developed a stronger sense of dignity, thus we can try to satisfy their needs and wishes, helping them to achieve dignified death with no regret.” (S2)* |
| 53 | P-FBDT could enhance emotional connections in family. (U) | | *“The P-FBDT could be an effective communication prompt for children and families to exchange their feelings and love with each other, which might have been ignored, or hard to express in daily life.” (S9)* |
| 54 | P-FBDT could relieve family grief in bereavement. (U) | | *“The love for each other they felt in the therapeutic process could potentially help the bereaved family to recover from the grief of losing a child, and the generativity entity could comfort the whole family through bereavement.” (S12)* |
| 55 | P-FBDT could enrich pediatric palliative care practice. (U) | | *“Dignity is the central goal of palliative care, however, there are few practical interventions to conserve the dignity of terminal children. P-FBDT with distinctive children-specific characteristics is different from DT for adults, which could enrich the practice of pediatric palliative care.” (S4)* |
| 56 | Difficult initiation of P-FBDT with children’s families. (U) | | *“The initiation of P-FBDT depends on the family’s acceptance of the diagnosis of the child. Families tend to insist on searching for possible treatments to cure their child, even if the disease is incurable. It is hard to initiate these topics with them in such a situation.” (S13)* |
| 57 | The uncertainty of the children’s compliance to P-FBDT. (U) | | *“It is more challenging to implement P-FBDT among younger children with underdeveloped understanding; they may lack concentration during the therapeutic process. Older children may be too rebellious to cooperate with their families or therapists.” (S6)* |
| 58 | Emotional challenges of participating in P-FBDT. (U) | | *“The life of advanced cancer children is incomplete so they might have much regret in life. While recalling the happiness in the past, the contrast to the present sufferings may cause emotional fluctuation in children and families.” (S13)*  *“Implementing P-FBDT might bring emotional challenges to the therapists. They may be empathetic to the sufferings of children and families, and need to maintain an intense attentiveness to cope with unexpected events during the session.” (S3)* |
| 59 | Therapists need to establish the trust relationships between the child, family, and therapist. (U) | | *“Therapists need to build good relationships with the children and their families by accompanying and understanding them, being empathic with their emotions, and helping to meet their practical needs.” (S9)* |
| 60 | Sufficient preparations before implementing P-FBDT. (U) | | *“The therapists should communicate with the children’s family to get familiar with their psychological and physical conditions, family relationships, religious beliefs, and so on, helping them to build a basic life framework for the family and make contingency plans for implementing P-FBDT.” (S6)*  *“The precondition of implementing P-FBDT includes adequate symptoms control as physical comfort is the foundation for satisfying other needs.” (S2)* |
| 61 | Flexible use of the P-FBDT protocol in practice. (U) | | *“Some adolescents are sensitive to their parents’ emotions, and they may be reluctant to express themselves in the presence of their parents. In that case, therapists can interview the child alone. The forms of implementing P-FBDT could be flexible and follow the wishes of participants.” (S7)*  *“During the P-FBDT interview, there may be a series of unexpected events such as emotional fluctuations of children or families, thus the P-FBDT session cannot be completed in 60 minutes. It is possible to conduct several times of sessions.” (S3)* |
| **Love et al. (2022). Bereaved parent perspectives and recommendations on best practices for legacy interventions.** | | | |
| Findings | | | Illustration |
| 62 | Legacy activities or items helped parents to make sense of their child’s illness or death. (U) | | Legacy item itself served as a tangible reminder of the purpose of their child’s illness experience: *“I know what the fight was for, I know why it happened; so, I’m a little eased when I see [the legacy item].”*  Post-death legacy activities helped parents find meaning and focus on the impact of their child’s life on others: *“We're trying to keep his memory alive by doing things like that. Helping other people. It kind of helps us with our grief while we can help others...we do a scholarship in his name and they help other kids' families that are going through the same thing.”* |
| 63 | Legacy items helped parents continue to feel connected to their child. (U) | | Legacy facilitated a physical, tangible bond between parent and child: *“So it still up to this day motivates me to keep going, and I say it’s like as if my baby is here laying on me. I put his teddy bear on my chest and lay there and I just I listen.”*  A legacy item helped to keep the child’s presence close to the parent: *“It just kind of makes me think of him during the day when I have [the fingerprint charm] on.”*  Legacy items connect parents with their child and provide solace during difficult moments: *“I’m like, ‘This is T-Man’s hand,’ and that’s how I can go about the day with it. If I’m missing him so much, I can go back to that [hand mold].”* |
| 64 | Legacy interventions offered concrete evidence that a child had once lived. (U) | | *“It’s like, ‘Listen to this [heartbeat song], look.’ It was proof she was here, here’s her heart. Look at this picture, look at this.”*  Affirmations of life were closely linked with a desire for others to remember the child: *“Other times, [I] am glad to have [the legacy items] because I want her to be remembered and know that I have to remember that she was here.”* |
| 65 | The parents’ intention in positioning legacy items in their homes was either to make them visible and accessible or to protect them. (U) | | Displaying items (e.g., curio cabinet) or keeping legacy items in view (e.g., framed pictures, jewelry charms) served as visible and tangible reminded for parents of their children. *“If I don't have my necklace on, I always really wear it, but I feel lost if I don't have it on.”*  Others safeguarded legacy items by keeping items in boxes, drawers, or other out-of-view areas: *“So for me it’s just still − it’s in the box it came in, and it’s just sitting there...I don't want anything to happen to it.”* |
| 66 | Legacy items over time to support parents’ ongoing grieving processes. (U) | | Parents described legacy items as a tangible anchor across the evolving grief trajectory, allowing them to return to the memory of their child and affirming their life: *“I can touch and see his fingerprint and push and hear the heartbeat...so it's like I see him, my baby, right here.”*  Parents reflected on how legacy items provided a grounding object for family, friends and community members to remain connected with the child: “The kids give him kisses all the time. They grab my necklace and give him kisses, or they’ll walk past the fridge and see his pictures and they give him kisses.”  Legacy items brought solace and relief to some parents: *“It’s wonderful to know that people hold these pieces of him, it disseminates him, and it takes the burden off of you as a parent.”* |
| 67 | Legacy items facilitated an ongoing relationship, or continuing bonds, with a child who has died, offering a concrete way to experience the child’s role in their life. (U) | | *“It helps me a lot because I don't want to just forget about her...[to] be able to look up and see all of her pictures on the wall, it puts a smile on, at least knowing that she can always be there.”* |
| 68 | In-depth and transparent communication were needed in the legacy-building process. (U) | | *“I think there might be better ways of presenting what the options are.”*  *“That can be a little uncomfortable, being like, ‘oh my child life specialist says,’ and you don’t want to ever feel like, ‘why did [that other family] get this’ or...[not know] what the criteria is.”* |
| 69 | Late introduction of legacy interventions can cause parents to negatively associate the legacy item with the child’s death. (U) | | *“I don’t necessarily want to always be reminded of the death moments, which a lot of the legacy items do because of how they’re produced tend to be associated with that actual end of life process.”* |
| 70 | Introduction of legacy interventions during the acute end-of-life process can intensify already hectic and stressful experience. (U) | | *“There’s a certain point where you’re like, oh my God. Oh, his breathing got really weird, oh no...we need to start morphine and we need to do these things, like oh my God...I would constantly − like we would think, okay, this is it, he’s about to die, and he didn’t die...It’s really hard to kind of relax into doing a little project.”*  *“They came into the room and I have to tell you honestly at that time I was like, ‘Are you f***ing kidding me?’ I'm sorry, and that's exactly how I felt.”* |
| 71 | Introduction of legacy interventions early in illness course may impair parents’ ability to continue hoping for a cure. (U) | | *“You know, I don't know that if they had told us about it previously. You wouldn’t because they knew it would be providing no hope to anyone. Like, hey, we got this if your son passes − well, no one is going to say that, and nor should they.”* |
| 72 | Legacy interventions feel more appropriate after all curative options have been exhausted and the child is certain to die. (U) | | *“You know it's going to come [the point when no further curative options existed]. And so when the moment came, she pulled me to the side and said, ‘Listen, we’re going to put him to sleep, and I want to see if you suggest to do a fingerprint and a heartbeat,’ she said, ‘so you can always have that.’”* |
| 73 | Creativity was a crucial element of individualized care. It was important to integrate unique or special patient attributes, personal memories, or family relationships into the legacy-building process. (U) | | One parent explained why her child’s handprint legacy item was uniquely special for her: *“The reason I wanted it was because she used to hold my hand when she wanted to go to sleep or anything like that. That was a big thing. She had to have a hold of your hand.”*  Parents also emphasized how important it was to engage the patients themselves in the process to create a truly individualized legacy item: *“[If you] wait a long time all you get is a heartbeat because that’s literally all that person can do. When they did it, it was a different juncture and having him singing along with the heartbeat with the song was incredible.”*  Parents recognized that each patient and family is unique, and achieving creative person-centered care in the context of legacy interventions requires thoughtful inquiry into each patient’s and family’s goals and preferences: *“[It’s] always helpful for people who make suggestions of things that work for them because every family is really different.”* |
| **Schaefer et al. (2020). Healing the hearts of bereaved parents: impact of legacy artwork on grief in pediatric oncology. Journal of Pain and Symptom Management. 60(4):790-800.** | | | |
| Findings | | | Illustration |
| 74 | Creating legacy artwork facilitates family bonding and memory making and opens lines of communication between family members regarding the child's impending death. (U) | | *“The daddy/mommy/me handprint legacy artwork was very meaningful because it was all of us working on that art as a family. We were creating memories as a family. So that helps to fill a small void in my heart, knowing we have those memories as a family with him.”—Parent*  *“There was a teen who was really depressed, and there were a lot of cultural things surrounding death that made it difficult for him to engage openly and honestly about his concerns about dying to his family. Creating a legacy artwork project (e.g., mural of a knight wearing a coat of armor and holding a shield) as a family allowed an opportunity for his parents to begin opening up and having conversations with him about what it means to protect others from difficult news and how to allow others to enter your space and become a little more vulnerable.”—Healthcare provider*  *“There was a teen who was really depressed, and there were a lot of cultural things surrounding death that made it difficult for him to engage openly and honestly about his concerns about dying to his family. Creating a legacy artwork project (e.g., mural of a knight wearing a coat of armor and holding a shield) as a family allowed an opportunity for his parents to begin opening up and having conversations with him about what it means to protect others from difficult news and how to allow others to enter your space and become a little more vulnerable.”—Healthcare provider*  *“Just like children who express themselves through play, art has the potential to open families up to deeper conversations. If you're spending time working on a piece of art together, it may open the door to some important conversations. Oftentimes, families and patients want to have those conversations, but they do not know how to start them. They may be difficult conversations, but they are conversations that need to happen.”—Healthcare provider*  *“We felt like creating the legacy artwork allowed us to talk about tough things without feeling pressured. It helped us put our feelings out there and to express ourselves. Sometimes, you just cannot put your pain into words. So instead of burying them, we used the art to share them with each other.”—Parent* |
| 75 | Legacy artwork provides opportunities for parents to engage in life review and meaning making of the child’s death. (U) | | *“Art is a representation of who we are in that moment, and who we are is our identity. And so, art is a reflection of the child, their identity. With words, we can fake. We can kind of tell our alternate story of who we want to be and we can wear masks sometimes, but with art, it very quickly strips away the masks, and the legacy becomes, you know, a child giving a story, an image, creating a sculpture, that’s completely reflective of who they are, and it’s demonstrated through the process of how it was created. Art is a reflection of the joy. A reflection of life, and because it is a creation, it is the antithesis to death and dying. And so for families, along with that joy, there is meaning that is created and that meaning moves through grief whether it is anticipatory or post-loss of their child.”—Healthcare provider*  *“We finished the original Wizard of Oz book tonight at bedtime. It said, ‘I realize that for my entire life, I had missed the point of the story, and it really hits home.’ As my voice broke, I read about how badly Dorothy wanted to get home. She never asked to be thrown into a new world in such a violent way. It was just a whirlwind that threw her around. It dropped her into a scary place that she knew nothing about. For us, that whirlwind is cancer. What she did not realize until the very end is that it mattered. Without Dorothy's journey, the scarecrow would have never gotten off that pole in the cornfield. The tinman never would've realized his potential for love. And the lion would've never embraced his power and strength. No matter where you are, if you're looking around and wondering, ‘Why me?’ and you're scared to death, it matters who knows how many lives have been changed because of your journey. Just keep pushing on. Everyone gets home in the end.’ The point of the Wizard of Oz painting was – it's the yellow brick road, that's heaven. That's the foundation of my belief in where she's going. God sent an angel to take my kid. That's what that tells me. It's okay.”—Parent*  *“We chose gold for her handprint on the family tree. It was for childhood cancer, but it was also for who she was as a child. It was shiny, and that's how she was. She was meant to shine. That's who she was and who she will always be no matter if she is with us or not. We knew her light would just keep on shining.”—Parent*  *“When you look at it, all you see is his legacy. It speaks volumes. You see love. You see sunshine. You see the kind of kid he was. You can tell that the only thing on his mind that day was not that he was sick. It was not that he was in the hospital. It was just that he was sitting in his room with his mommy and it mattered what was going on. He felt safe, happy, and warm. But the painting also shows that I'm leaving you. It shows that maybe he knew something about himself that I did not know and that maybe God had talked to him.”—Parent*  *“The legacy artwork showed that our family is an everlasting circle. Her handprints in the middle of ours represented us surrounding her with love, but at the same time, the butterfly she made with her handprints meant that we were giving her the freedom to fly, take her wings, and go to heaven to be with God. This painting expresses what our family was like in our last moment as one but also tells me that we will all be together again someday.”—Parent* |
| 76 | After the child’s death, parents display the legacy artwork in their home and take comfort in using these projects to continue their bond with their deceased child. (U) | | *“We definitely have it somewhere special and obvious, you know, out where everyone can see it that comes into our house. We want people to admire it.”—Parent*  *“The legacy was that after her daughter died, the mom took the entire wall mural home and put it in her bedroom. Now the art piece continues to have an existence somewhere else, and it has a name and it has a story.”—Healthcare provider*  *“And then in the end, um, he signed it, with a paint pen, and um, there's actually a texture to it cause he went into the paint with it. And so, I remember him painting it. I remember him signing it, you know. I always touch it. And I always kind of run my fingers across where he signed it, where he tagged it. And that just … ahh, there's something about like a tag tile, you know. I do not know why, you know, but I know that he wrote it, I know that he did it, and it takes me back to him. Sorry, I'm choking up.”—Parent*  *“I'll put my hands on the glass sometimes on top of his small handprints, and I'll feel like I'm holding him again. His sisters do the same thing. Even though one is only two, she knows that those are brother's handprints, and she loves to go touch them. It's comforting for the whole family.”—Parent*  *“He would always give me crap about being a Georgia fan. And he would do his Daddy the same about being an Alabama fan. I could be having a really bad day and I can walk through and see his Auburn-themed canvas, and I'm like ‘look, he's still picking at me even afterward.’ It just warms my heart knowing he is still with me.”—Parent*  *“The art legacy is that it is tangible, that it is something the families can hold onto. These pieces the families chose to take with them, chose to keep, chose to frame, and chose to hang up in their spaces are a continual reminder of the connection and the relationship between them and their child. And so legacy artwork offers an opportunity for parents to feel like their child is still teaching them, still giving them something, and still with them.”—Healthcare provider*  *“These legacy artwork pieces help families have something to hold in their hand when they walk out the door. You know, something that they can feel like they are taking a piece of their children with them and where they can feel a sense of closeness to the patient. One of the hardest things I've seen families do is just not really knowing how to walk out of the hospital without their patient, but these legacy artwork projects allow them to take a piece of their child home with them.”—Healthcare provider* |
| 77 | The experience of participating in legacy artwork ameliorated parents’ grief after the death of their child to cancer. (U) | | *“Well at first when he passed, it was hard for me to look at his artwork, but then I realized this should not make me sad. It brings back happy memories, and I will just take his art piece off the wall, and I’ll sit there and hold it and look at it and think of the good memories. It has helped me move through my grief journey.”—Parent*  *“We attach positivity to it when we use it as a part of our child’s memorial service or in an ongoing way, and we continue to attach meaning to it. With meaning, there is hope. There is um this knowledge that life is never going to be the same. Um, but the grief is profound, and it’s real and it’s hard and it’s deep. Um, but it offers us a little bit of hope in reminding us that it was a life well lived--that they were here, that they did make this, and that it was not something that was just made up. And there is hope in that. You know, there is hope in the masses.”—Parent*  *“The family circle artwork brings me a peace. When I look at it all these years later, I just feel kind of a sense of peace about her death. So, I definitely think, especially when we are having a hard time, I think it means a whole lot to all of us.”—Parent*  *“I'm a mechanical engineer. I've been doing it for 30 years. So, I am very pragmatic, and it's almost as if to get through this loss, you have to shed so many tears [crying], and every time I do that, it feels better. It helps me cope with the loss of my son. So, when I see his artwork, I cry, and that helps me get through.”—Parent*  *“That piece reminds me that there's more out there. It reminds me of those special moments in life, so it definitely influences the way I process my grief and the way I see life. It makes each day just a little bit easier.”—Parent* |
| 78 | Legacy artwork may reduce compassion fatigue and burnout among healthcare providers as well as provide an outlet for coping with the death of their patients. (U) | | *“Care providers do experience their own grief and loss. So, we would conduct a ‘Refresh and Restore’ session every now and then where we would invite the care team to come together. I remember once we painted trees, and we worked with the metaphor of trees because, you know, trees you could deeply root, but then they also go through the seasons of change. It was very restorative with the care team to have their own experience to create and to remember the lives that they have cared for.”—Healthcare provider*  *“When care teams start to get to a point where you know, the next death is just another deathdwhen you start to see that there is some post-trauma that begins to accumulate, and it’s worsened with the loss, then art can be a wonderful and powerful intervention to have them be a part of.”—Healthcare provider*  *“It was an important way for us to honor her while she was still here and to show our support and also a little bit of personal expression of our grief for her. Through this project, she developed really meaningful relationships with her care team, and when we think back to that, we can remind ourselves of the special and important moments we shared, even though the outcome was not what anybody wanted.”—Healthcare provider* |
| 79 | Legacy artwork should be offered as early and frequently as possible and to all children with cancer to mark their illness journey rather than reserving it as an end-of-life project. (U) | | *“I mean I personally think the earlier the better. We do not have to make it seem like this doom and gloom sort of thing that you know, ‘Oh, well you better do this now because you’re never going to get an opportunity again to remember your child.’ If we wait too long, then it suddenly becomes the death project. Whereas you know, giving it, framing it as something that is going to give you guys the opportunity or a chance to do something together to make these wonderful memories and regardless of the outcomes, you’re going to have this beautiful thing to mark this moment of your life, of your journey.”—Healthcare provider*  *“I would not want a person to come in and tell me, ‘oh well, you do not know how long you may have her so we are going to do legacy work.’ I would have been angry because I still had hope. I think saying ‘This is just a part of her story and this is just going to be a way for you to mark this time of her story’ would be more helpful and comforting.”—Parent*  *“I love the legacy work we can do when the child is awake and alert and interactive opposed to the legacy projects when the child can no longer participate. I worry that if we wait too long, we solidify a memory for families of when the child has died or is dying. And I think there’s a tendency to idolize, for point of a better word, the dying child instead of the child in these moments.”—Parent*  *“If we all agree that this is hugely important for the child's processing as well as the parents' grief and bereavement, then it should be just as important as all those other things. Right? It's not just this afterthought. It's not just a nice thing that we do for these kids that are sick, but it's an integral part of their care plan.”—Healthcare provider* |
| 80 | It is important to communicate the benefits of legacy-making with members of the medical team who are less familiar with complementary supports. (U) | | *“I think there is a lot of value in having buy-in from the team. If an oncologist states ‘part of your care is going to be using art as a tool for exploration,’ there is a lot of weight that comes from him/her validating this activity. Then families may go, ‘Oh, this might be as important as some of the other modalities of care that are being offered.”—Healthcare provider* |
| 81 | Parents suggested hiring a full-time art therapist, as opposed to a contracted position, to increase availability for families. (U) | | *“It would be cool if they had a full-time art therapist, you know, somebody who was very much involved in the day-to-day, knows what we have been through that week. I think the experience would be so much richer if there was a full-time person doing legacy work and other types of art therapy.”—Parent* |
| 82 | Parents wished for the hospital to host legacy art shows to commemorate their children's lives. (U) | | *“In terms of legacy, his artwork enables me to be proud and to talk about him and to brag about him. Because like what I've shown you, look how proud I am! To have our children's legacy artwork formally recognized in an art show or an auction, I think that would make all of the difference in the world for us as parents.”—Parent* |
| **Akard et al. (2021). Long-term follow-up of legacy services offered by children's hospitals in the United States. Palliative Medicine Reports. 2(1):218-225.** | | | |
| Findings | | | Illustration |
| 83 | Children’s hospitals staffs suggested the need to determine potential benefits of legacy services to patients and their family members and health care providers. (U) | | For example, one participant suggested research to explore the ‘*‘benefit of legacy work prior to the death, when the child is still able to participate.’’*  Others suggested examination of *“‘benefits of legacy materials/activities for families’ and ‘effects on siblings.’”*  One participant suggested research including potential benefits of legacy services on health care providers: “*I would also like to know more about the impact of facilitating legacy and end of life projects on CCLS [certified child life specialists].”* |
| 84 | Children’s hospitals staffs suggested research related to the long-term effects of legacy activities. (U) | | For example, one participant suggested “*follow-up with families sometime after the death of a child (six months, a year, etc.) and ask them if they were offered legacy items, did they participate, and if so, has it helped with the grieving process.”* |
| 85 | Children’s hospitals staffs suggested research related to the best time to introduce legacy services. (U) | | Six participants wanted research to enhance our understanding for *“when is the best time to introduce [legacy] services.”* |
| 86 | Children’s hospitals staffs emphasized that the mechanism of intervention effect is based on the legacy-building process rather than the product. (U) | | One participant said, *“This experience is about process not product. all the items play the same role in supporting the family.”*  Another participant shared, *“The memory is in the doing, rather than in the creation of a thing. The thing is the product of an amazing experience together and a reminder of that time.”* |
| **Foster et al. (2012). National survey of children's hospitals on legacy-making activities. Journal of Palliative Medicine. 15(5):573-8.** | | | |
| Findings | | Illustration | |
| 87 | Children’s hospitals staffs suggested the need for further research and dissemination of knowledge on legacy-making activities. (N) | A child life director suggested long-term follow-up studies on families and siblings. Another participant recommended that researchers find out what parents say about how the legacy-making activity helped them through their ordeal. Some participants desired more information and ideas about legacy-making activities, while others would like to know what others are doing. Another suggested more surveys of those who offer this service and conferences on this topic. | |
| 88 | Children’s hospitals staffs suggested offering legacy-making activities sooner in the illness trajectory. (U) | One participant said, *“A lot of people tend to wait until the child is near death. It is important that this is done as a standard of care from the beginning of diagnosis.”*  One respondent recommended legacy activities for *“earlier in the process so they can fully participate.”* Another said, *“It would be interesting to see if it makes a bigger impact on the child and family if you offered legacy-making activities earlier versus later. For example, when the child is first diagnosed versus when they are (receiving) palliative care.”*  One participant added, *“It isn’t always end of life, but sometimes saying goodbye to some of themselves, literally. In one case, a teen patient had to have a leg amputation. So our staff helped this boy to cast a mold of his leg.”*  Another participant recommended *“not always looking at them (legacy-making activities) from the ‘after death’ perspective. These activities can be fantastic for a patient who survives to look back on as tangible evidence of their psychosocial and physical journey.”* | |
| 89 | Children’s hospitals staffs suggested an increase in staff education in regards to legacy-making. (U) | *“Offer education for professionals so that they can better understand how to approach sensitive situations such as these.”*  Another participant shared, *“Legacy-making can be important throughout a serious illness.but the medical team.is not always ready for some of these activities.”*  Another reflected, *“I wish that all staff were better at encouraging and helping families participate in legacy-making, rather than just doing it themselves.”*  One participant suggested potential benefit to staff: *“[It’s] something useful for staff who worked with the patient and family who has died or (is) dying. Especially those that die after being hospitalized for a long time with the regular staff on a day-to-day basis. It’s hard on them.”* | |
| 90 | Children’s hospitals staffs suggested the need to individualize legacy-making activities for each child and his or her family. (U) | One participant shared, *“It is important to offer families the option of participation – being present when legacy-making activities are being created, or having legacy materials created not in their presence. Sharing with families that their choice will be respected.”*  Another participant commented on the balance between standardizing and individualizing these services: *“We are working.to standardize how/when we offer to each patient or family, while being able to individualize.”*  Others suggested, *“Be flexible, offer a variety of activities”and “find out as much as you can about the child and family, what is important to them.”*  One participant suggested, *“Just taking the cues from the children as to how much they want to do or if they want to do it.”* | |

**Supplementary Material 4 – Results of Meta-Aggregation of Qualitative Findings**

Results of meta-aggregation of qualitative studies and qualitative components of mixed methods studies findings. These synthesized topics were derived from 90 study findings that were assigned as “Unequivocal” and “Credible” and were aggregated into 12 categories.

| **Synthesized findings** | **Category** | **Findings** |
| --- | --- | --- |
| Perceptions about legacy-making interventions | Positive attitudes for legacy-making interventions | 1.Children perceived the Digital Storytelling intervention as fun and enjoyable. (U) (Finding 1)  2.Parents expressed positive comments and appreciation for the legacy intervention. (U) (Finding 3)  3.Parents liked the Web-Based intervention exactly as it was. (U) (Finding 6)  4.All parents were positive about the movie format of legacy intervention, specifically mentioning their appreciation for the coherent and automated flow of the entire story. (U) (Finding 13)  5.Parents found the legacy building items to be a positive experience, and reported finding great meaning in the objects. (U) (Finding 37)  6.The term “Legacy Building” wad comforting and reflected parents’ positive experiences. (U) (Finding 42) |
|  | Challenges with legacy-making interventions | 1. Difficult initiation of P-FBDT with children’s families. (U) (Finding 56)  2. The uncertainty of the children’s compliance to P-FBDT. (U) (Finding 57)  3. Emotional challenges of participating in P-FBDT. (U) (Finding 58) |
| Legacy-making experiences of children and family members | Legacy-making experience of children | 1.Many children living with advanced cancer did things to be remembered, including making crafts for others, willing away belongings, writing letters to loved ones, and giving special gifts. (U) (Finding 16)  2.Children did not intentionally do or say things to be remembered because of their illness, age, or unexpected death. Because some children with advanced cancer did not know they were dying, or family members think their child with cancer did not need to do or say anything to be remembered. (U) (Finding 17)  3.All children expressed gratitude to their families and/or friends in the legacy videography. (U) (Finding 23)  4.Some children gave instructions in the legacy videography about who should have some of their personal items after their death. (U) (Finding 24)  5.Children recalled positive memories in the legacy videography. (U) (Finding 25)  6.Children made comments related to death/dying, as well as to missing family, friends, or experiences in the legacy videography. (U) (Finding 26)  7.Children described some of their disease experience as well as the stigma of disease in the legacy videography. (U) (Finding 27)  8.Spiritual statements were made by some children reflecting spirituality as a source of comfort in the legacy videography. (U) (Finding 28) |
|  | Remembrance and legacy-making experience of family members | 1.Bereaved family members remembered the deceased child. They remembered the qualities of the deceased child. (U) (Finding 18)  2.Children’s concern for family was remembered by family members. (U) (Finding 19)  3.Children’s beliefs about an afterlife was remembered by family members. (U) (Finding 20)  4.For some families, the most meaningful aspect of legacy building is in the events or projects that they create in memory or honor of their child. (U) (Finding 40)  5.One further aspect of legacy building was the personal connections made with hospital staff while the child was alive. (U) (Finding 41)  6.Parents remembered their child through touching objects, having legacy building items visible in the home, and through the public and private ways they have honored their child’s life. (U) (Finding 43)  7.Parents’ therapeutic and personal relationship with various members of the medical team influenced the memory that parents had to the legacy items. (U) (Finding 46)  8.The parents’ intention in positioning legacy items in their homes was either to make them visible and accessible or to protect them. (U) (Finding 65)  9.After the child’s death, parents display the legacy artwork in their home and take comfort in using these projects to continue their bond with their deceased child. (U) (Finding 76)  10.Parents never being offered legacy building ,or not being explicitly told what legacy building was or the intended purpose for legacy building. They felt anger and regret for that. (U) (Finding 30)  11.Legacy objects made after a child had died brought negative experience for parents. (U) (Finding 34)  12.Parents felt that the legacy building objects were not helpful, or harmful, for their grief, or not meaningful. The disappointment or negative experience was in the quality of the objects provided to them. (U) (Finding 35)  13.Parents did not like the term “Legacy Building”. (U) (Finding 36)  14.The objects provided and made by hospital staff were infused with significantly less meaning compared to objects made by family or friends, or bought or created by the parents themselves in remembrance of their child. (U) (Finding 38)  15.Siblings disliked looking at items such as photos or tangible reminders of their sibling. (U) (Finding 50) |
| Impact of legacy-making interventions | Positive impacts of legacy-making interventions | 1.Parents perceived positive intervention benefits for both the children and their family who were involved in creating their child’s story, including emotional expression and communication benefits and parent-child relationship benefits. (U) (Finding 4)  2.Parents perceived the new movie feature of legacy intervention facilitated family conversations. (U) (Finding 14)  3.Families elaborated the heartbeat recordings were meaningful for preserving of memories with their child. (U) (Finding 15)  4.Children’s personal experience of living with cancer inspired the children to affect the lives of others. Cancer inspired children to prepare themselves for death or prepare others for their death. (U) (Finding 21)  5.While cancer inspired children with cancer to live their lives differently, their advice, in turn, inspired bereaved parents and siblings to live life differently in the moment. (U) (Finding 22)  6.Altruism helped bereaved parents /caregivers honor and carry on their child’s legacy. The experience of losing a child has helped them channel their grief into serving others. (U) (Finding 29)  7.One meaningful aspect of legacy building was opportunities to continue their child’s legacy and memory after their death. (U) (Finding 39)  8.Tangible items created by the child, or created with the child, were important as representing a proof of life. Tangible items kept the relationship and connection in the present as opposed to the past. (U) (Finding 44)  9.Parents’ relationship to the legacy objects and items has shifted and changed over the course of their grief. (U) (Finding 45)  10.Looking at the legacy building items brought back positive memories for siblings. (U) (Finding 48)  11.Legacy building items helped siblings cope with and express their grief. (U) (Finding 51)  12.Legacy activities or items helped parents to make sense of their child’s illness or death. (U) (Finding 62)  13.Legacy items helped parents continue to feel connected to their child. (U) (Finding 63)  14.Legacy interventions offered concrete evidence that a child had once lived. (U) (Finding 64)  15.Legacy items over time to support parents’ ongoing grieving processes. (U) (Finding 66)  16.Legacy items facilitated an ongoing relationship, or continuing bonds, with a child who has died, offering a concrete way to experience the child’s role in their life. (U) (Finding 67)  17.Creating legacy artwork facilitates family bonding and memory making and opens communication regarding the child’s impending death. (U) (Finding 74)  18.Legacy artwork provides opportunities for parents to engage in life review and meaning making of the child’s death. (U) (Finding 75)  19.The experience of participating in legacy artwork ameliorated parents’ grief after the death of their child to cancer. (U) (Finding 77)  20.Legacy artwork may reduce compassion fatigue and burnout among healthcare providers as well as provide an outlet for coping with the death of their patients. (U) (Finding 78)  21. P-FBDT could facilitate dignity conservation for terminal children. (U) (Finding 52)  22. P-FBDT could enhance emotional connections in family. (U) (Finding 53)  23. P-FBDT could relieve family grief in bereavement. (U) (Finding 54)  24. P-FBDT could enrich pediatric palliative care practice. (U) (Finding 55)  25. Children’s hospitals staffs emphasized that the mechanism of intervention effect is based on the legacy-building process rather than the product. (U) (Finding 86) |
| Suggestions for improvement | Individual needs consideration in legacy-making interventions | 1.Parents suggested that more should be taken into account to accommodate the individual needs of ill children in future legacy intervention. (U) (Finding 8)  2.Creativity was a crucial element of individualized care. It was important to integrate unique or special patient attributes, personal memories, or family relationships into the legacy-building process. (U) (Finding 73)  3.Children’s hospitals staffs suggested the need to individualize legacy-making activities for each child and his or her family. (U) (Finding 90) |
|  | Family members should be included in legacy-making interventions | 1.Parents suggested to involve family members more in future legacy intervention. (U) (Finding 7)  2.Parents wanted to have had the choice to participate in the creation of legacy building items if  the opportunity would have been given to them. (U) (Finding 31)  3.Siblings thought it’s important to have the opportunity for the whole family to make legacy building items. (U) (Finding 49) |
|  | Timing for offering legacy-making interventions | 1.Parents’ suggestions regarding the best timing for when to offer the intervention ranged from the beginning to end of the illness trajectory. (U) (Finding 5)  2.Parents felt that the legacy intervention program should be lengthened to start sooner and last longer. (U) (Finding 9)  3.It was important to offer legacy building sooner and more often throughout a child’s illness or hospitalization. (U) (Finding 32)  4.It was important for parents to have the opportunity to create legacy building items while their child is alive. (U) (Finding 47)  5.Late introduction of legacy interventions can cause parents to negatively associate the legacy item with the child’s death. (U) (Finding 69)  6.Introduction of legacy interventions during the acute end-of-life process can intensify already hectic and stressful experience. (U) (Finding 70)  7.Introduction of legacy interventions early in illness course may impair parents’ ability to continue hoping for a cure. (U) (Finding 71)  8.Legacy interventions feel more appropriate after all curative options have been exhausted and the child is certain to die. (U) (Finding 72)  9.Legacy artwork should be offered as early and frequently as possible and to all children with cancer to mark their illness journey rather than reserving it as an end-of-life project. (U) (Finding 79)  10.Children’s hospitals staffs suggested offering legacy-making activities sooner in the illness trajectory. (U) (Finding 88)  11.Children’s hospitals staffs suggested research related to the best time to introduce legacy services. (U) (Finding 85) |
|  | The intervention protocol should be improved | 1.The most frequent suggestion from parents was to make the web program more user-friendly. (U) (Finding 11)  2.Parents had suggestions for improved intervention content and design. (U) (Finding 12)  3.Sufficient preparations before implementing P-FBDT. (U) (Finding 60)  4.Flexible use of the P-FBDT protocol in practice. (U) (Finding 61) |
|  | Suggestions related to well-trained multidisciplinary intervention team | 1.The best person on the psychosocial team to initiate legacy conversations and also offer more opportunities for legacy building would be the child life specialist. (U) (Finding 33)  2.Therapists need to establish the trust relationships between the child, family, and therapist. (U) (Finding 59)  3.In-depth and transparent communication were needed in the legacy-building process. (U)(Finding 68)  4.It is important to communicate the benefits of legacy-making with members of the medical team who are less familiar with complementary supports. (C) (Finding 80)  5.Parents suggested hiring a full-time art therapist, as opposed to a contracted position, to increase availability for families. (U) (Finding 81)  6.Children’s hospitals staffs suggested an increase in staff education in regards to legacy-making. (U) (Finding 89) |
|  | Suggestions related to creating a sharing platform | 1.Parents desired a sharing or community component to allow the children to read others kids’ stories and increase their awareness of other patients. (U) (Finding 10)  2.Parents wished for the hospital to host legacy art shows to commemorate their children's lives. (U) (Finding 82) |
|  | Suggestions related to the short- and long-term impacts of legacy services | 1.Children’s hospitals staffs suggested the need to determine potential benefits of legacy services to patients and their family members and health care providers. (U) (Finding 83)  2.Children’s hospitals staffs suggested research related to the long-term effects of legacy activities. (U) (Finding 84) |
